# Grhl3 promotes retention of epidermal cells under endocytic stress to maintain epidermal architecture in zebrafish

**DOI:** 10.1371/journal.pgen.1009823

**Published:** 2021-09-27

**Authors:** Mandar Phatak, Shruti Kulkarni, Lee B. Miles, Nazma Anjum, Sebastian Dworkin, Mahendra Sonawane

**Affiliations:** 1 Department of Biological Sciences, Tata Institute of Fundamental Research, Mumbai, India; 2 Department of Physiology, Anatomy and Microbiology, La Trobe University, Bundoora, Australia; 3 Center for Biotechnology, A.C. College of Technology, Anna University, Chennai, India; Cologne University, GERMANY

## Abstract

Epithelia such as epidermis cover large surfaces and are crucial for survival. Maintenance of tissue homeostasis by balancing cell proliferation, cell size, and cell extrusion ensures epidermal integrity. Although the mechanisms of cell extrusion are better understood, how epithelial cells that round up under developmental or perturbed genetic conditions are reintegrated in the epithelium to maintain homeostasis remains unclear. Here, we performed live imaging in zebrafish embryos to show that epidermal cells that round up due to membrane homeostasis defects in the absence of *goosepimples/myosinVb (myoVb)* function, are reintegrated into the epithelium. Transcriptome analysis and genetic interaction studies suggest that the transcription factor Grainyhead-like 3 (Grhl3) induces the retention of rounded cells by regulating E-cadherin levels. Moreover, Grhl3 facilitates the survival of MyoVb deficient embryos by regulating cell adhesion, cell retention, and epidermal architecture. Our analyses have unraveled a mechanism of retention of rounded cells and its importance in epithelial homeostasis.

## Author summary

Developing vertebrate epidermis isolates and protects growing embryos from their surroundings. For performing such a crucial function under compromised physiological or genetic conditions, robust mechanisms allowing maintenance of epidermal integrity are warranted. However, such mechanisms are not fully explored. To investigate the mechanisms by which epidermis copes up with drastic cell-shape changes to maintain the epidermal integrity, we have used a mutant condition, *goosepimples/myosinVb* (*myoVb)*, wherein epidermal cells round up due to defective intracellular membrane trafficking. Our in vivo confocal imaging shows that this cell rounding is transient and the rounded cells are not extruded. Instead, they are retained and reintegrated. Using next generation sequencing and in situ expression analyses, we show that *grainyhead-like 3* (*grhl3*) gene as well as several cell adhesion genes, including *e-cadherin* (*cdh1*), are up-regulated in the epidermal regions having rounded cells. Our genetic analyses reveal that the function of *grhl3*, which encodes for a transcription factor shown to be crucial for epidermal differentiation and wound healing, is essential to retain rounded-up cells by increasing E-cadherin mediated cell adhesion. We further show that this retention is essential for the maintenance of epidermal homeostasis. We propose that such a mechanism may be operational whenever cells round up under developmental or perturbed genetic conditions.

## Introduction

Epidermis is an epithelial tissue that forms the outermost covering in metazoans. In vertebrates, it is stratified and consists of multiple layers of tightly packed keratinocytes that are held together by adherens junctions and desmosomes [1]. In addition, tight junctions present in the granular layer prevent trans-epidermal water loss [2, 3], whereas focal adhesions and hemidesmosomes mediate the adhesion between epidermis and basement membrane [4–7]. This stratified organization develops from a bilayered epidermis having similar junctional organization [8, 9]. The robust bilayered architecture allows the epidermis to function as a living barrier during early vertebrate development to protect the highly vulnerable milieu interior, thereby ensuring proper development and survival.

Transcription factors belonging to the highly conserved Grainyhead family act as important regulators of epidermal morphogenesis, wound healing, and barrier function [10–16]. Consistent with this, the three mammalian *Grainyhead-like* (*Grhl*) paralogs control the expression of genes important for cell adhesion and extracellular barrier repair. *Grhl1*, *Grhl2*, and *Grhl3* control the expression of Desmoglein 1 (Dsg1) (Wilanowski et al., 2008), E-cadherin and Claudin-4 [17, 18]and Transglutaminase 1 (TGase 1), respectively [19–21]. In mice, *Grhl3* is particularly important for epidermal proliferation, differentiation, and maintenance of epidermal barrier function [19, 22]. *Grhl3* regulates miR-21, which is implicated in skin diseases such as squamous cell carcinoma and atopic dermatitis [23, 24]. Moreover, whereas a GRHL3-dependent pathway is essential for suppression of disease initiation and accelerated healing of lesions in immune mediated hyperplasia, loss of GRHL3 leads to upregulation of the chemokine TARC and increased epidermal inflammation [25, 26]. In *Xenopus*, *Grhl3* promotes the fate of superficial layer of the bilayered embryonic epidermis [27, 28], whereas it regulates epiboly in zebrafish [28–30]. The increase in EVL/peridermal cell surface area–in the absence of any other effect on EVL/peridermal fate, integrity and adhesion—has also been reported in *grhl3* morphant embryos at early developing stages though the reason for this increase has remained unclear [30].

Shape and size of cells are two major parameters influencing the architecture as well as homeostasis of epithelial tissues and are tightly controlled. Cell-cell and cell-matrix adhesions are important regulators of cell shape dynamics [31]. Whereas increased cadherin based adhesion promotes polygonal cell morphology, its mis-regulation promotes cell-rounding [32–34]. Transient cell rounding is a common phenomenon in epithelial tissues under various physiological conditions. It is a prerequisite for morphogenetic processes [35, 36] and is observed during epithelial mitosis [37, 38]. However, the persistence of rounded up cells in epithelia leads to defective tissue topology and cell crowding, resulting in their basal extrusion (delamination) or apical extrusion [39–42]. Recently, it has been shown that during wound healing, stretch activated channels sense local crowding to promote homeostatic cell extrusion, thereby maintaining cell density [43]. Nevertheless, cell rounding is not always associated with extrusion. As reported in various *Drosophila* epithelia, misalignment of mitotic spindle plane–induced by ectopic expression of *inscuteable*–produces misplaced rounded cells. Interestingly, these cells are reintegrated into the epithelial sheet, which requires lateral adhesion mediated by Neuroglian and Fasciclin-2 [44]. However, it is not clear whether such mechanisms are present in vertebrate epithelia to reintegrate rounded up cells arising due to perturbed genetic or physiological conditions.

The early developing bilayered epidermis in zebrafish consists of an outer peridermal layer and underlying basal epidermis. It is an ideal system to investigate mechanisms essential for maintaining epithelial homeostasis. Previously, using two mutant loci, *goosepimples* (*gsp/myoVb*) and *romeharsha (rhs)*, a direct link between plasma membrane homeostasis, cell size maintenance, and cell proliferation has been demonstrated [45, 46]. In addition, it has been reported that peridermal cells round up in *gsp/myoVb* and *rhs* mutant embryos, presumably due to perturbed membrane homeostasis [45, 46]. The fate of these rounded up cells and the consequences of their retention or extrusion on tissue architecture have not been investigated so far.

Our *in vivo* imaging analysis presented here reveals that rounded up peridermal cells in MyoVb deficient embryos are not extruded; instead, most of them are retained and reintegrated. Moreover, in the absence of *myoVb* function, epidermal regions bearing rounded up cells show enhanced *grhl3* expression, which is essential for cell retention and maintenance of epidermal homeostasis. Mechanistically, *grhl3* promotes retention of rounded up peridermal cells in MyoVb deficient embryos by regulating E-cadherin mediated cell-cell adhesion.

## Results

### Peridermal cells, rounded up in the absence of *gsp/myoVb* function, are retained and reintegrated to facilitate phenotypic recovery

**Fig 1:**
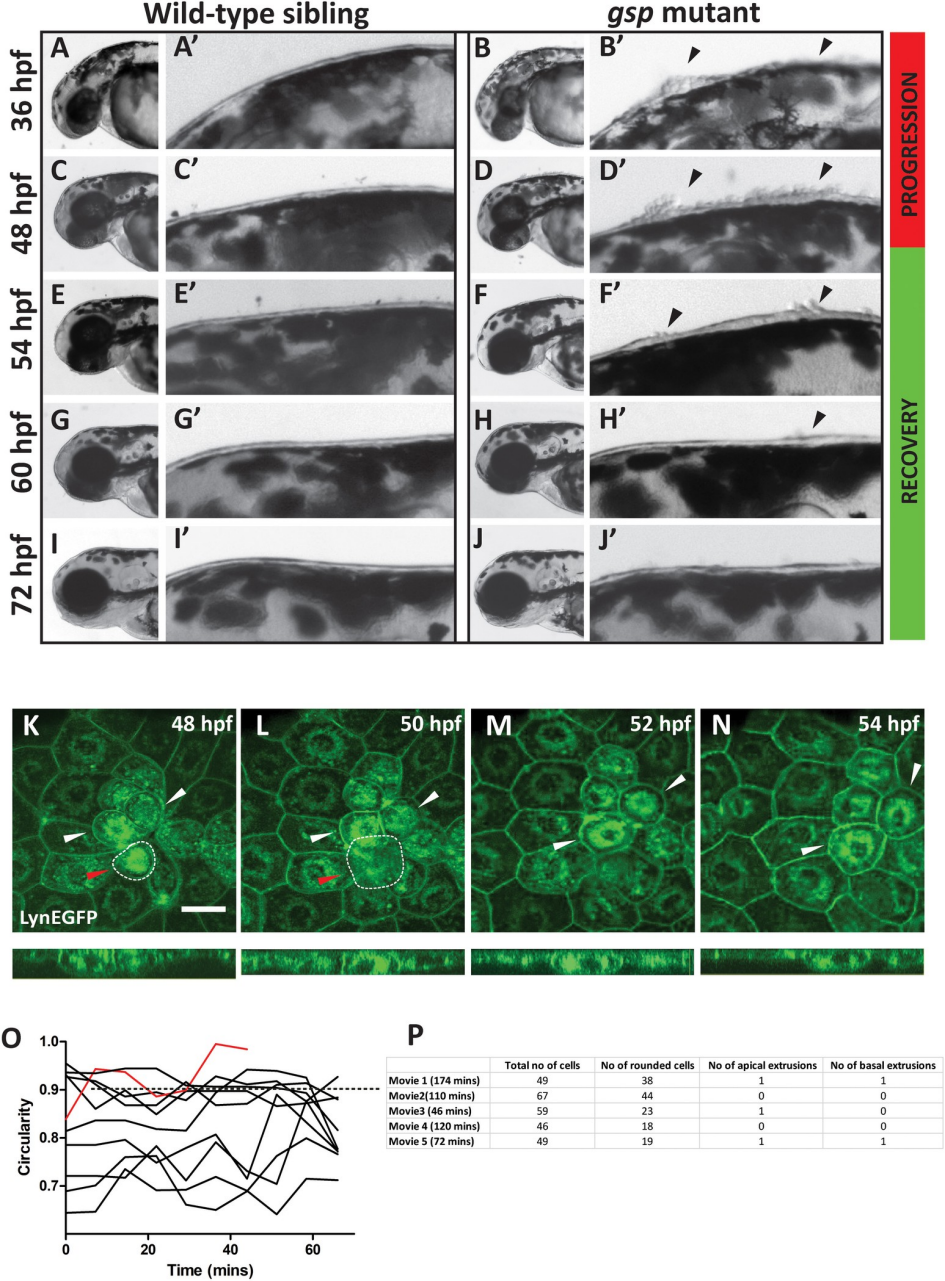
Regression of peridermal cell rounding phenotype occurs through reintegration of rounded cells. DIC images of individual wild-type sibling (A, C, E, G, I) and *gsp* mutant (B, D, F, H, J) embryos imaged successively at 36, 48, 54, 60, and 72 hpf, respectively. A’–J’ represent the corresponding enlarged images of dorsal head epidermis. Arrowheads indicate regions showing cell rounding. Note that cell rounding increases until 48 hpf (progression) and decreases subsequently (recovery) to finally cease by 72 hpf. Confocal time lapse images of dorsal head epidermis of cldnB:lynEGFP embryos injected with *myoVb* splice site morpholino (*myoVb* MO) during the recovery period at 48 (K), 50 (L), 52 (M), and 54 (N) hpf along with respective orthogonal projections. All cell borders as well as several intracellular membrane compartments are marked by lynEGFP (green). White arrows represent reintegrated cells, showing progressive cell shape change from round to polygonal. Red arrow and white dotted outline indicate a rounded cell undergoing apical extrusion. Scale bar = 50 μm. Traces showing changes in cell circularity values (O) for ten randomly selected cells in the above movie. Red trace represents extruding cell. Dotted line represents cell circularity = 0.9, which was used as the benchmark for classifying cells as rounded. A table (P) showing total number of cells, number of rounded cells, apical extrusions and basal extrusions (delamination) in live confocal movies of *myoVb* morphant embryos made between 50-53hpf. The duration of the movie is indicated in parentheses.

In a forward genetic mutagenesis screen aimed at identifying genes important for the maintenance of epidermal integrity and architecture, zebrafish mutants showing transient rounding up of epidermal cells, namely *goosepimples* (*gsp*) and *romeharsha* (*rhs*), were isolated [46, 47]. Although it is known that the *gsp* locus encodes for Myosin Vb, an actin based molecular motor [45], the molecular nature of *rhs* has remained unknown. The *gsp* as well as *rhs* mutant embryos exhibit a profound defect in intracellular transport–characterized by increased endocytosis and perinuclear accumulation of early, recycling, and late endosomes–leading to perturbed membrane homeostasis in the peridermal cells. Consequently, surface area of the individual peridermal cell reduces and proliferation in the epidermis increases. Additionally, several peridermal cells, especially over the head, exhibit rounding up [45, 46]. We have earlier shown that these rounded up cells project out of the plane of the periderm [45, 46]. To better characterize the cell rounding phenotype, we carefully observed the head epidermis of individual *gsp/myoVb* mutant embryos between 36 to 72 hours post fertilization (hpf). Although the peridermal cell rounding phenotype was most profound at 48 hpf, it started subsiding thereafter, and cell rounding was absent by 72 hpf (Fig 1A–1J’).

Given that peridermal cell rounding phenotype is transient in *gsp/myoVb* mutant embryos, we asked whether disappearance of rounded up cells results from cell shedding or cell reintegration. We used a specific splice site morpholino (*myoVb* MO) to deplete Myosin Vb levels in embryos derived from cldnB:lynEGFP transgenic line, in which peridermal cell membranes are marked by lynEGFP [45, 48]). Like *gsp/myoVb* mutant embryos, embryos injected with *myoVb* MO developed a fully penetrant phenotype with almost every embryo beginning to develop clumps of the rounded up peridermal cells on the head between 36 to 48 hpf. Live confocal imaging of the *myoVb* morphant periderm in the dorsal head region between 48 hpf and 54 hpf revealed that most of the rounded up peridermal cells were retained in the epidermis (Fig 1K–1N and S1 Movie) and a few got reintegrated. The cell-reintegration was assessed based on visual inspection of shape transition from rounded to polygonal in X-Y plane and from more round to squamous morphology in X-Z plane (Fig 1K–1N). The analysis of cell circularity on fixed samples (presented in Fig 4U) revealed that in wild type embryos peridermal cells usually do not show circularity values above 0.9, which can be used as a threshold for classifying rounded cells. We analyzed circularities of selected cells during recovery phase in the live movie (S1 Movie), which revealed that cells with circularity higher than 0.9 persisted in the epithelium (Fig 1O). Additionally, we followed rounded cells in five movies that were made between 50 hpf to 53 hpf. We observed that apical cell-extrusion and basal cell extrusion (delamination) events were observed rarely (Fig 1P). As reported earlier [45], the accumulation of perinuclear vesicles persisted even after the phenotypic recovery (Fig 1N).

To conclude, the regression of peridermal cell-rounding phenotype occurred not through extrusion of rounded cells, but rather through their retention and re-integration.

### Comparative transcriptomics reveals upregulation of *grhl3* and cell adhesion genes in *gsp* and *rhs* mutant embryos

**Fig 2:**
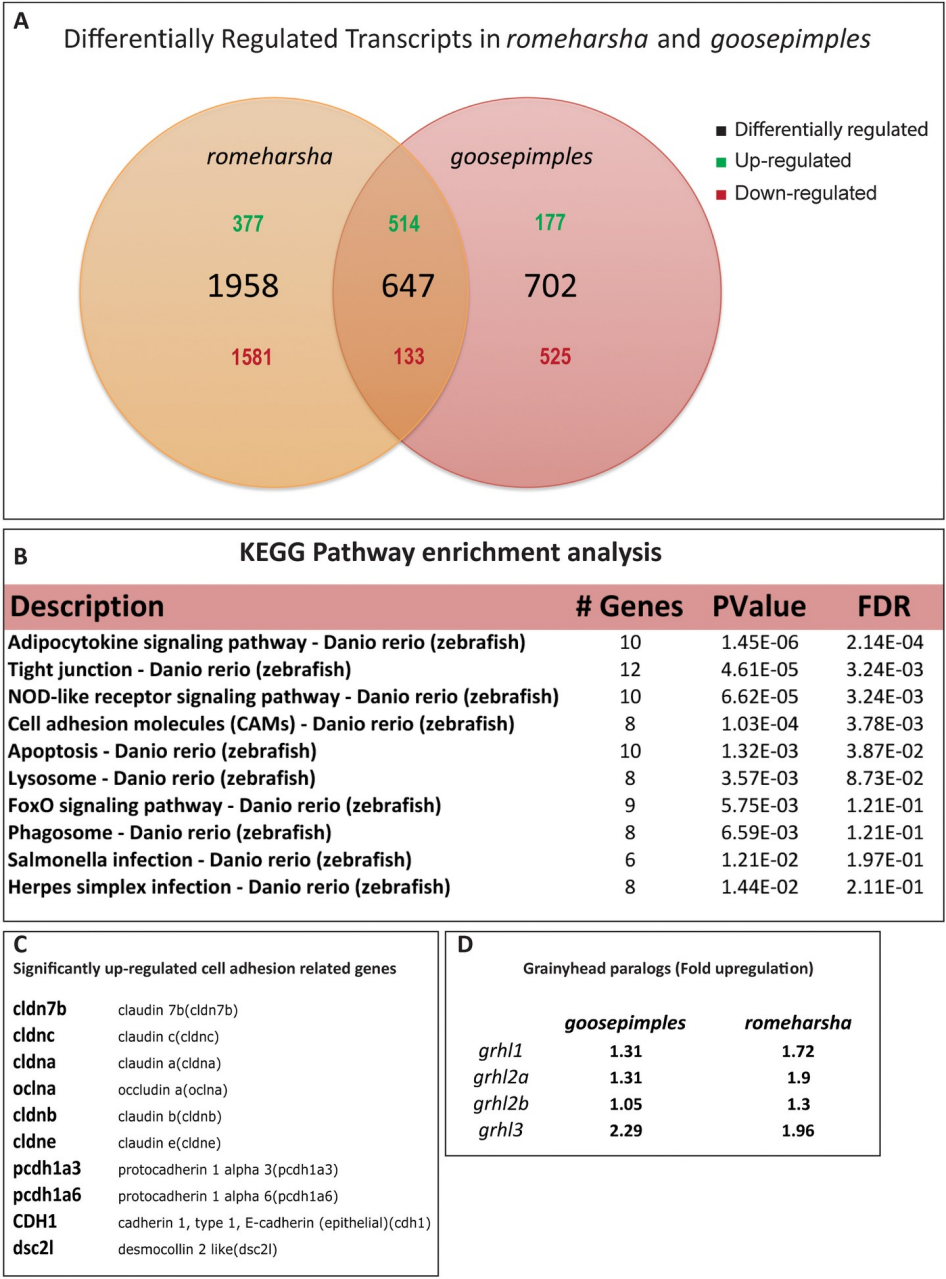
Comparative transcriptomics analysis of *gsp* and *rhs* reveals upregulation of *grhl3* and adhesion related genes. Venn diagram showing the numbers of differentially regulated transcripts in *gsp* and *rhs* (A). Pathway analysis of differentially regulated genes, common to both the genetic conditions, using KEGG pathways (B). Significantly upregulated cell-adhesion genes common to *gsp* and *rhs* (C). Fold upregulation of different *grainyheadlike* paralogs in *gsp* and *rhs* (D).

As shown above, the degree of peridermal cell rounding was the highest at 48 hpf, and reduced thereafter due to reintegration of rounded up cells. This analysis suggested that at 48 hpf, the factors responsible for retention and reintegration of rounded up cells begin to confer their effect. To screen for genes that might be responsible for retention and reintegration of rounded-up cells, we performed transcriptome analysis of *gsp/myoVb* and *rhs* embryos at 48 hpf using RNAseq. We reasoned that the use of *rhs* mutant embryos, which also exhibit peridermal cell rounding due to intracellular transport defects [46], would allow identification of the factors that are specifically involved in the epidermal response. Total mRNA was isolated from 50 *gsp/myoVb* and *rhs* mutant embryos and their wild-type siblings at 48 hpf, reverse transcribed, and sequenced using Illumina Hiseq 2000 (GEO accession: GSE173649). We obtained 1349 and 2605 differentially regulated transcripts in *gsp* and *rhs* mutants, respectively (S1 Data). We omitted transcripts whose expression was differentially regulated exclusively in either of the mutants and retained only those that were significantly up/downregulated to a similar degree in both the mutants. We obtained 514 and 133 transcripts that were up- and down-regulated, respectively, in both the mutants with 1.48-fold (1.5 σ) as the threshold (Fig 2A). Over Representation Analysis (ORA) using KEGG pathways revealed upregulation of transcripts associated with adipocytokine pathway, foxo pathway, tight junctions, and cell adhesion (P < 10^−4^) (Fig 2B). The cell adhesion associated genes included those encoding for desmosomal, adherens junction, and tight junction components (Fig 2C). We also observed that amongst the transcription factors, the transcripts for *grainyhead like 3* (*grhl3*) were upregulated in both *gsp* and *rhs* mutant embryos (Fig 2D). This was interesting, given the importance of Grainyhead family-transcription factors in epidermal differentiation and regulation of cell adhesion components. Importantly, while *grainyhead* paralogs *grhl1* and *grhl2a* were also significantly upregulated in *rhs* mutant embryos, only *grhl3* showed upregulation above the set threshold of 1.48-fold in the *gsp/myoVb* mutants (Fig 2D).

To conclude, the transcriptomic approach allowed us to identify genes that are differentially regulated in mutants showing epidermal cell rounding phenotype arising due to defects in intracellular transport. Upregulation of cell adhesion components and *grhl3* may be causally linked with the restoration of epidermal architecture in the *gsp* mutant embryos.

### Expression of *grhl3* is spatiotemporally coincident with epidermal cell-rounding in *gsp/myoVb* deficient embryos

**Fig 3:**
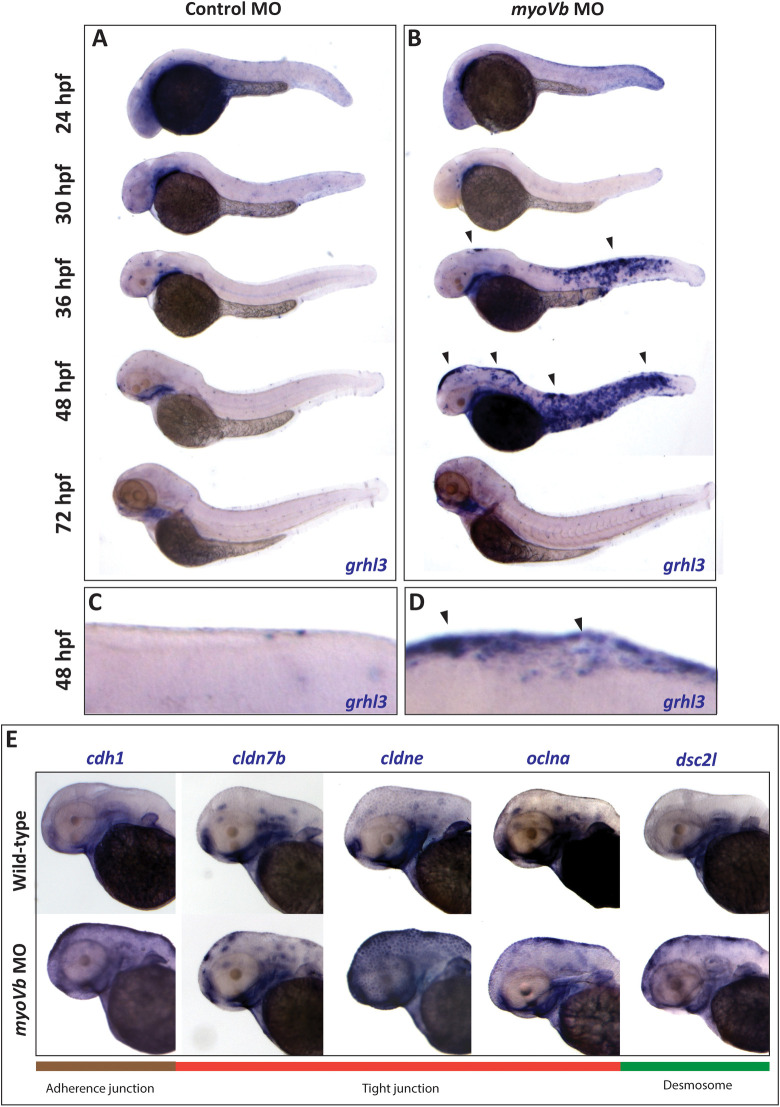
Whole mount RNA *in situ* hybridization (WISH) reveals spatiotemporal association between cell rounding, *grhl3* expression, and expression of cell adhesion genes. Bright field images of control morphant (A) and *myoVb* morphant (B) embryos at 24, 30, 36, 48, and 72 hpf stained for *grhl3* transcripts. Zoomed in images of dorsal head epidermis of control morphant (C) and *myoVb* morphant embryos (D) stained for *grhl3* expression at 48 hpf. The *grhl3* expression was observed near the epidermal regions showing cell rounding (black arrowheads). WISH reveals increased expression of cell adhesion genes, *cdh1*, *cldn7b*, *cldne*, *oclna*, and *dsc2l*, in and around the epidermal regions containing rounded cells in *myoVb* morphants at 48 hpf (E).

The process of peridermal cell-rounding and reintegration occurs during a restricted time window. Given the role of Grainyhead transcription factors during epidermis development, we probed the expression of *grhl3*, using whole mount in situ hybridization (WISH), at 24, 36, 48, and 72 hpf. As shown earlier [30, 49], control zebrafish embryos showed low levels of *grhl3* expression in the epidermis at 24 hpf, which diminished by 36 hpf. After 30 hpf, the expression mostly remained restricted to the pharynx and nasal placodes (Fig 3A). The *grhl3* expression pattern in the *myoVb* morphant epidermis at 24 hpf was similar to that of controls, and a visible decrease in the expression was evident in the head epidermis at 30 hpf. However, *grhl3* expression reappeared specifically in the aggregates of rounded up cells in the epidermis of the *myoVb* morphants at 36 hpf and persisted at 48 hpf (Figs 3A, 3B, S2A and S2B). By 72 hpf, g*rhl3* expression in MyoVb deficient embryos was highly reduced and comparable to that in the controls.

As mentioned earlier, comparative transcriptome analysis of *gsp* and *rhs* revealed up-regulation of genes encoding for tight junction components and other cell adhesion proteins. We performed WISH to assess the expression patterns of the adherens junction component *cadherin1* (*cdh1*), tight junction components *claudin 7b* (*cldn7b*), *claudin e* (*cldne*), and *occludin a* (*oclna*), and desmosomal component *desmocollin2 like* (*dsc2l*), in *myoVb* MO and control MO injected embryos. Whereas control embryos showed minimal expression of these genes in the epidermis at 48 hpf, MyoVb deficient embryos showed increased expression in and around the aggregates of rounded-up peridermal cells (Fig 3E).

To conclude, our expression analysis revealed that *grhl3* expression is spatiotemporally coincident with cell rounding in the MyoVb deficient embryos. Moreover, the adhesion components *cdh1*, *cldn7b*, *cldne*, *oclna*, *and dsc2l* showed elevated expression specifically in and around the rounded up peridermal cells, suggesting their potential involvement in cell retention and re-integration.

### *grhl3* function is essential for retaining peridermal cells in MyoVb deficient embryos

**Fig 4:**
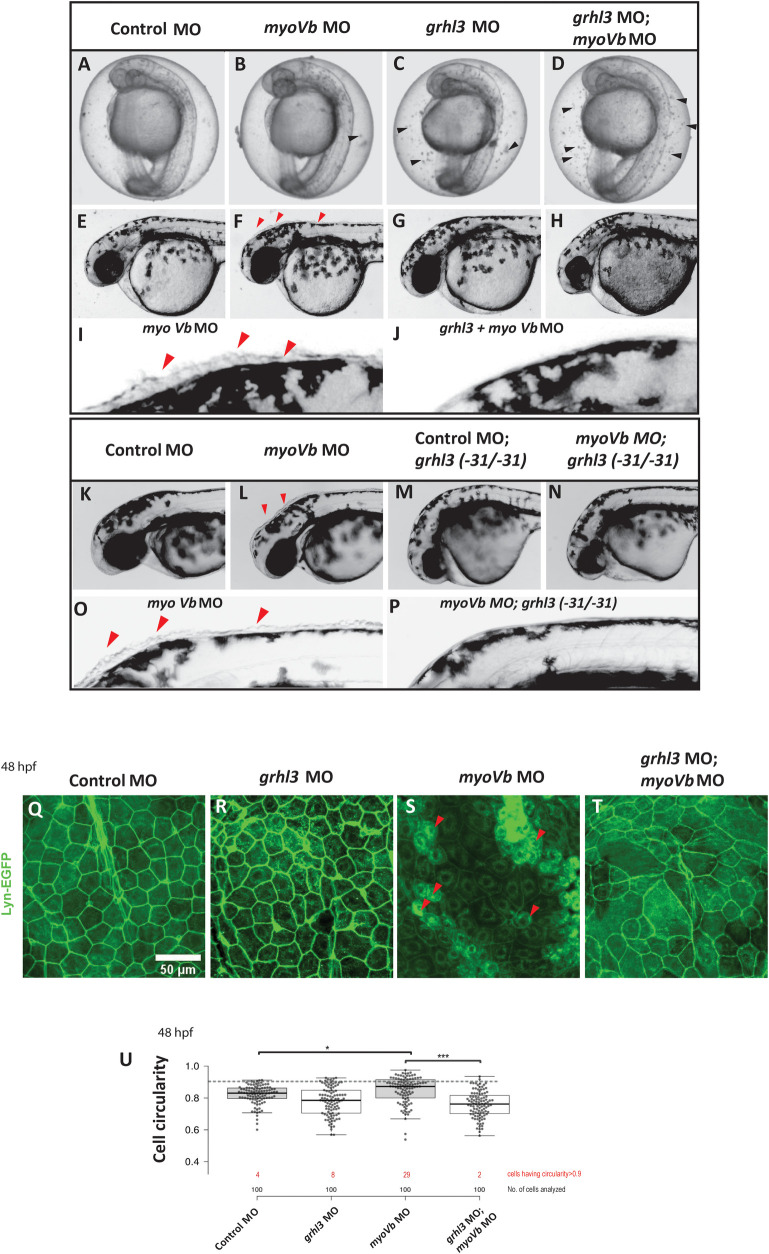
Simultaneous knockdown of *grhl3* and *myoVb* shows absence of cell rounding. Bright field images of embryos at 28 hpf injected with control MO (A), *myoVb* MO (B), *grhl3* MO (C), and *grhl3* MO; *myoVb* MO (D). Black arrowheads indicate cell debris accumulated inside the chorion of *grhl3* and *grhl3;myoVb* double morphant embryos, presumably due to epidermal cell shedding. Representative images of the head region of zebrafish larvae injected with control MO (E), *myoVb* MO (F), *grhl3* MO (G), and *grhl3;myoVb* double morphant (H) at 36 hpf. Zoomed in images of the dorsal head region of *myoVb* morphant (I) and *grhl3;myoVb* double morphant (J) embryos, respectively. Representative images of wild-type siblings injected with control MO (K) and *myoVb* MO (L) and *grhl3*(-31/-31) mutants injected with control MO (M) and *myoVb* MO (N). Zoomed in images of dorsal head region of wild-type siblings (O) and *grhl3* (-31/-31) mutants (P) injected with *myoVb* MO. Note the presence of rounded cells in *myoVb* MO in F, I, L, O—indicated by red arrowheads—and their absence in *myoVb; grhl3* double deficient embryos in H, J, N, P. Representative confocal images of the peridermal cells from the stated genetic conditions at 48hpf (Q-T). Note the presence of rounded cells in *myoVb* MO indicated by red triangles. Box and whisker plot (U) showing quantitation of cell circularity in control, *grhl3* MO, *myoVb* MO, and *grhl3;myoVb* MO at 48hpf. Each column represents cell circularities of 25 cells each from four embryos per condition (total 100). Grey dotted line represents the cell circularity threshold used for classifying round cells. Red numbers indicate % cells having cell circularity values above threshold. *myoVb* MO embryos show significantly higher median cell circularity than that in control and *grhl3;myoVb* MO (Kruskal Wallis One way ANOVA p<0.05). For all comparisons and statistical significance please refer to S2 Data.

Our expression analysis pointed towards a spatiotemporally regulated function of *grhl3* in MyoVb deficient embryos. We further explored whether *grhl3* has a function in the resolution of aggregates of rounded up peridermal cells. For this, we knocked down *grhl3* function in MyoVb deficient embryos, using a morpholino targeting the translation initiation site. The *grhl3* knockdown alone resulted in no obvious morphological phenotypes at 48 hpf (Fig 4G) except minor deformities in the median fin-fold (S1B Fig). As expected, MyoVb deficiency resulted in rounding-up of peridermal cells on the head. Interestingly, knockdown of *grhl3* function in *myoVb* morphants did not produce rounded up cells in the periderm at 36 hpf (Fig 4F, 4H, 4I and 4J). These embryos, deficient for both Grhl3 and MyoVb, showed significant lethality at 72 hpf as compared to that in either Grhl3 or MyoVb deficient embryos (S1A Fig). Similar lack of rounded up cells was seen in *gsp/myoVb* mutants injected with *grhl3* MO (S1C Fig). However, the epidermis showed irregular morphology (S1C Fig) and the embryos seemed fragile, as the yolk oozed out through the epidermis during mild perturbation such as manual dechorionation at 30 hpf. The *gsp/myoVb* mutant embryos deficient for Grhl3 died between 36–48 hpf. Since morpholinos have been reported to exhibit off-target effects, we verified these observations using additional morpholinos (S1A Fig) published previously; one targeted against translation initiation site and two against splice sites [28, 50]. To further confirm that the effect was specifically due to impaired *grhl3* function, we used a hypomorphic *grhl3* mutant allele generated using CRISPR-Cas9 technique. This hypomorphic *grhl3* (-31) allele, contains a 31 bp deletion in exon 4, which results in a frameshift to produce a truncated peptide of 224 aa lacking the conserved DNA binding domain (S1D Fig). The *grhl3* (-31/-31) mutant embryos complete epiboly, are embryonically viable, and exhibit impaired craniofacial development similar to that previously reported in *grhl3* morphants (S1E Fig; [50]). MyoVb deficiency in *grhl3* (-31/-31) mutant embryos resulted in the absence of rounded up peridermal cells that are characteristic of *gsp/myoVb* mutant or morphant embryos (Fig 4N and 4P). Analysis of cell circularity in fixed samples at 48hpf revealed that cells in *myoVb* MO epidermis showed a significantly higher median circularity as compared to that in control, *grhl3* MO and in *grhl3;myoVb* MO epidermis (Fig 4Q–4U, Kruskal Wallis one-way ANOVA with Dunn’s post hoc test, p<0.05). Importantly, in wild type embryos only 4% cells showed circularity value higher than 0.9 suggesting that cell circularity value occasionally exceeded beyond 0.9 in the normal epidermis. Using this as a threshold, we found that *myoVb* MO epidermis showed higher proportion of 29% cells with high circularity as compared to 8% in *grhl3*MO and 2% in *grhl3;myoVb* MO epidermis. These analyses suggested that Grhl3 expressing *myoVb* MO epidermis sustained a larger population of rounded cells.

**Fig 5:**
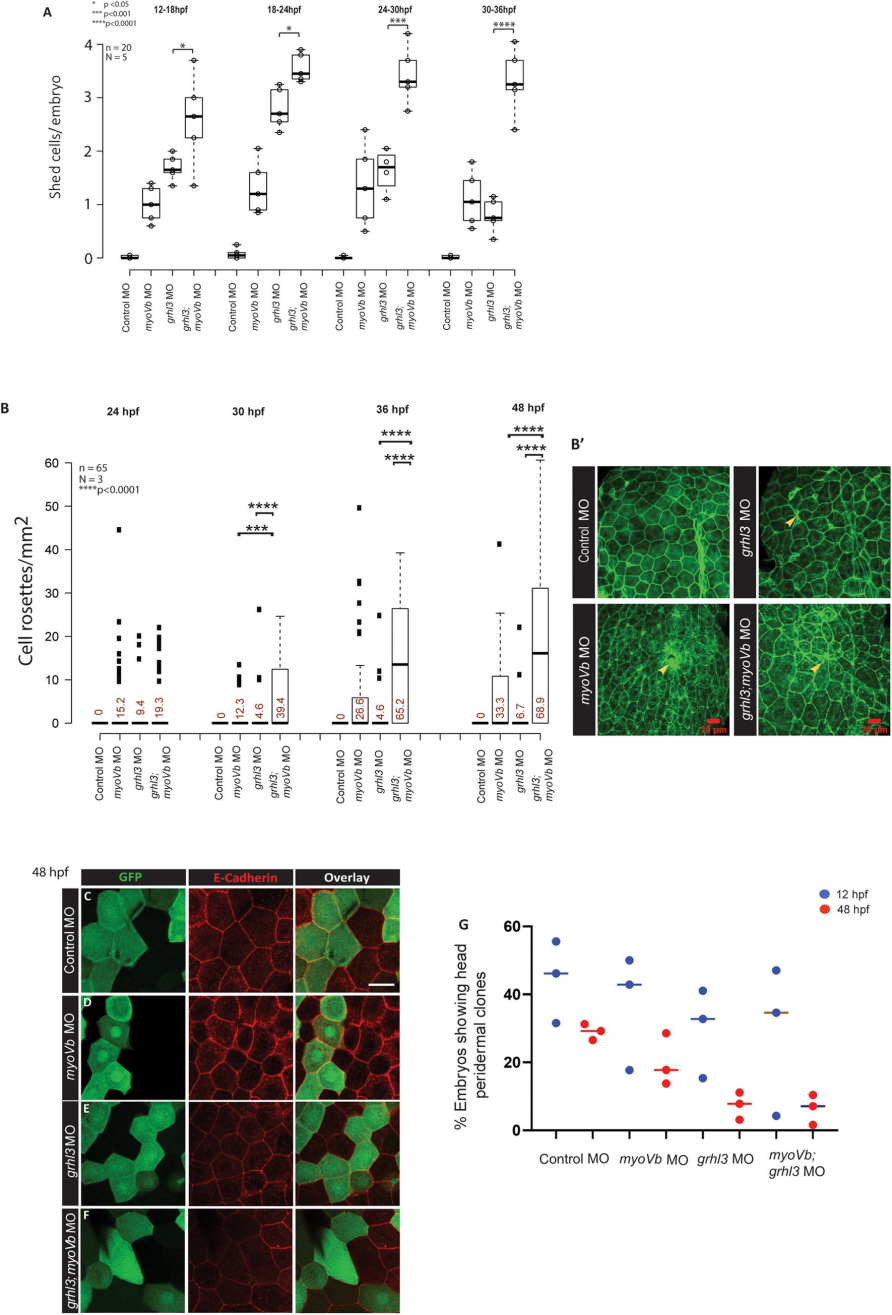
*grhl3* function is essential for cell retention in both wild-type and MyoVb deficient epidermis. A box and whisker plot (A) showing quantification of cell shedding by control morphant, *grhl3* morphant, *myoVb* morphant, and *grhl3;myoVb* double morphant embryos during time windows: 12–18 hpf, 18–24 hpf, 24–30 hpf, and 30–36 hpf. Note that post 24 hpf, cell shedding in *grhl3* morphants is comparable to that in *myoVb* morphants, whereas that in *grhl3;myovb* double morphants remains significantly higher (data presented as box plots showing median and IQR, n = 20, N = 5, one-way ANOVA, p<0.0001). Quantification of peridermal rosette structures in the dorsal head region. Box and whisker plot shows rosette densities (rosettes/mm^2^) at various developmental stages (B). Numbers in red indicate % embryos showing rosettes. Median rosette density is comparable at 26 hpf in all conditions. However, at 32 hpf, 36 hpf and 48 hpf, *grhl3;myoVb* morphants show significantly higher rosette density and occurrence than those in either *grhl3* or *myo*Vb morphants. Statistical significance was calculated using Kruskal Wallis one-way ANOVA with Dunn’s post hoc test. Representative confocal images (B’) showing rosettes indicated by yellow arrowheads in stated genetic conditions. Confocal images of dorsal head periderm showing clones injected with control MO, *myoVb* MO, *grhl3* MO, and *grhl3+myoVb* MO and marked by GFP (green) and stained for E-cadherin (red) at 48 hpf (C, D, E, F). Scale bar = 50 μm. The percentage of embryos showing peridermal clones was calculated as readout of cell retention probability in the periderm. The dot plot shows quantification of clone retention at 12 hpf and 48 hpf (G); individual points represent % embryos showing peridermal clones in a set, horizontal line denotes median. n ~ 60, N = 3. Note that *grhl3* and *grhl3;myoVb* double morphant clones show lower retention compared to those of control morphant or *myoVb* morphant clones at 48 hpf. Square brackets (in A and B) indicate comparisons and asterisks represent statistically significant difference. Only relevant comparisons in A and B are shown. For all comparisons and statistical significance please refer to S2 Data.

The absence of rounded-up peridermal cells in Grhl3;MyoVb double deficient periderm could be interpreted as either the suppression of cell-rounding or as extrusion and loss of the rounded-up cells. Careful observations during early development revealed that *grhl3* knockdown alone results in the accumulation of cell debris in the chorion of approximately 68% embryos at 28 hpf (S1A Fig). Interestingly, the extent of accumulation as well as number of embryos showing accumulation were increased in Grhl3;MyoVb double deficient embryos compared to those in all other conditions (Figs 4A–4D and S1A). However, only a small proportion (21.6 ±8%) of *myoVb* morphants exhibited this phenotype (Figs 4B and S1A). Consistently, *grhl3(-31/-31)* mutant embryos injected with control MO or *myoVb* MO showed accumulation of cell debris in the chorion (S1F Fig). Further temporal analysis revealed that the cell accumulation in *grhl3* morphants and *grhl3;myoVb* double morphants started becoming apparent at 28 hpf and increased progressively (S3A Fig). This accumulation of cells in the chorion suggested that epidermal cells are shed from the embryos in the absence of *grhl3*. To quantify the cell shedding directly, we pelleted down cells that were shed in E3 medium by dechorionated embryos in every 6 h time-window between 12–36 hpf. The pelleted cells were then shortly incubated in Trypan blue, and stained (dead) cells were counted. Quantification revealed that while *myoVb* morphants exhibited moderate cell shedding between 12 hpf to 24 hpf, *grhl3* morphants and *grhl3;myoVb* double morphants exhibited significantly higher cell shedding than that in *myoVb* morphants. Between 24 hpf to 36 hpf, *grhl3*;*myoVb* double morphants showed much higher cell shedding than that in *myoVb* and in *grhl3* morphants (Fig 5A, One-way ANOVA with Tukey’s test, p<0.05). Interestingly, this time window coincides with that of emergence of perinuclear vesicles (~24 hpf) and appearance of cell rounding (~36 hpf) in the MyoVb deficient embryos [45]. A similar increase in cell shedding was observed between 36 hpf and 42 hpf in Grhl3;MyoVb double deficient embryos (S3B Fig, One-way ANOVA with Tukey’s test, p<0.005). Corroborating this, live DIC imaging of Grhl3;MyoVb double deficient embryos mounted in 3% methyl cellulose at 36 hpf showed constant cell extrusion from the head epidermis (S3C Fig). Live confocal imaging of *grhl3;myoVb* double morphant embryos between 30 and 40 hpf suggested that the peridermal cells round-up before extrusion and that the extrusion events might trigger a cascade of extrusion of neighboring cells (S2 and S3 Movies). Such cascading effect was not observed in *myoVb* MO embryos though the cell extrusion events took place in densely packed patches of rounded cells (S4 Movie). Preliminary live analysis indicating the absence of extrusions (S5 Movie), along with the extensive rosette analysis presented below, suggested that the propensity towards extrusion from the head periderm was low in Grhl3 deficient embryos post 30 hpf. Further quantification of number of extrusions in three movies of *grhl3;myoVb* double morphants made between 50 to 53 hpf (S4E Fig) revealed that the number of rounded cells was quite low while the number of extruding cells was higher in these double morphants as compared to only *myoVb* morphants (Fig 1P). Live time course analysis of circularity in peridermal cells selected from four movies, including 4 representative cells that extruded in *myoVb* MO as well as in *grhl3;myoVb* MO embryos, revealed that extruding cells attain high circularity prior to their extrusion (S4A–S4D Fig) in both the genotypes.

Further analysis of cellular features by live confocal microscopy revealed that the loss of *grhl3* caused peridermal cells to exhibit basolateral membrane projections, which were more pronounced in the *grhl3;myoVb* double morphant periderm, indicative of compromised cell-cell adhesion (S3D and S3D′ Fig and S6–S8 Movies).

It was clear from live analysis that cell extrusion events are highly stochastic and therefore live analysis was not sufficient to allow useful comparisons between cell extrusion rates across conditions. Therefore, we quantified cell-rosettes—which form after cell extrusions [51, 52]—as proxies for extrusion events in fixed samples. Rosettes were defined as 5 or more cells converging at a single point. Despite their transient nature, rosettes were readily observed in *grhl3* MO, *myoVb* MO and *grhl3*;*myoVb* MO embryos (Fig 5B’), indicating that rosettes could be captured before their resolution. Quantification of rosettes at multiple time-points revealed higher number of embryos showing extrusions as well as significantly higher number of extrusion sites per unit area in *grhl3*;*myoVb* double deficient embryos than those in either *grhl3* or *myoVb* MO embryos beyond 30hpf (Fig 5B, Kruskal Wallis one-way ANOVA with Dunn’s post hoc test, p<0.0001). The near absence of cell-rosettes in the control embryos indicated that either the cell extrusions were absent or a few in number. It also suggested that developmental cell-rearrangements leading to rosette formation [53] were rare in the developing zebrafish epidermis.

Finally, we asked whether there is an increase in apoptosis in the absence of *myoVb* and *grhl3* function leading to extrusion of the peridermal cells. Anti- Activated Caspase 3 antibody staining at 26 hpf, 32 hpf, 36 hpf, and 48 hpf revealed that apoptotic cells were rarely present in the periderm under all the three genetic conditions. It appeared that only cells that were undergoing terminal phase of extrusion or delamination (basal extrusion) were apoptotic (S3E Fig). At 36 hpf, none of the 135 wild-type embryos analyzed exhibited any apoptotic peridermal cell. However, in MyoVb and Grhl3 deficient conditions we observed 3 apoptotic cells in 76 embryos and 1 apoptotic cell in 82 embryos, respectively. Interestingly, in 109 double deficient embryos, we did not observe any apoptotic cell presumably due to their rapid extrusion.

Our analysis indicated that *grhl3* is expressed specifically in the regions containing rounded-up peridermal cells. This points towards a region-specific requirement of *grhl3* in the retention of peridermal cells. To test this possibility, we injected *myoVb* MO, *grhl3* MO, and *grhl3+myoVb* MO along with EGFP mRNA as a tracer in wild-type embryos at 16–32 cell stage and calculated the proportion of embryos showing peridermal clones in the head region. Our analysis revealed that at 12 hpf, clone retention was comparable in all conditions; however, at 48 hpf, both Grhl3 deficient and Grhl3;MyoVb double deficient clones were less frequently detected than control and *myoVb* morphant clones (Fig 5C–5G). These data point towards a cell autonomous role of *grhl3* in promoting cell retention in the periderm.

To conclude, our data suggest that although *grhl3* function is important for preventing cell shedding during early epidermis development, its function becomes increasingly important in the absence of *myoVb*, where membrane homeostatic defects result in cell rounding.

### *grhl3* is important for maintaining the tissue architecture and homeostasis in the MyoVb deficient epidermis

Our data show that *grhl3* function is essential for prevention of epidermal cell extrusion and becomes more relevant in the absence of *myoVb* function, where endocytic stress and perturbed intracellular transport leads to decreased cell size and peridermal cell rounding [45, 46]. We further investigated the consequences of the increased cell extrusion in the MyoVb deficient epidermis.

**Fig 6:**
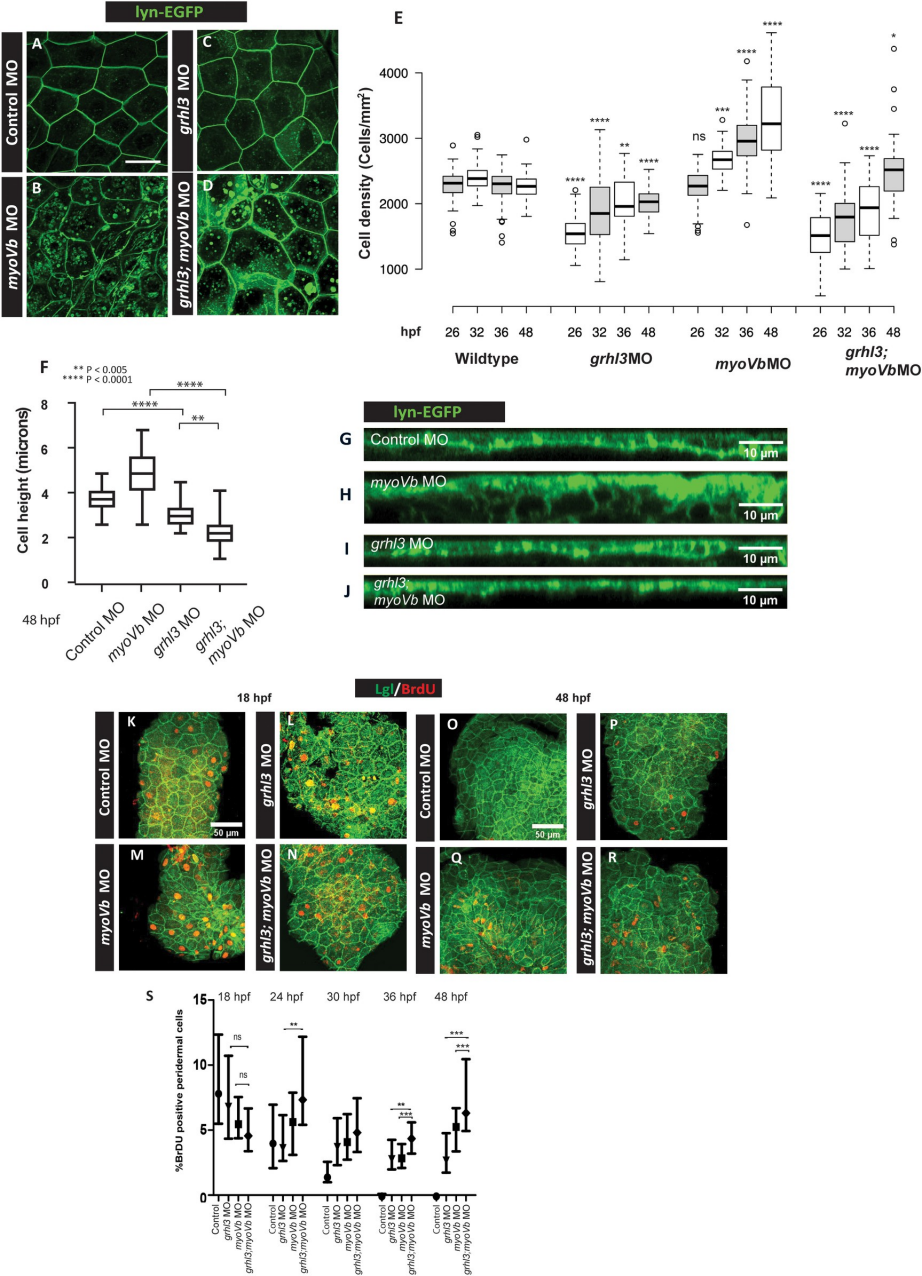
Cell loss due to Grhl3 deficiency leads to altered tissue architecture and homeostasis. Representative confocal images of cldnB:lynEGFP embryos (A-D), injected with control MO, *myoVb* MO, *grhl3* MO, and *grhl3* MO; *myoVb* MO. Peridermal cell density (cells/mm^2^) at different time-points in given genetic conditions (E). Note that *grhl3* morphants as well as *grhl3*;*myoVb* morphants show lower cell density than wildtype at 26 hpf. However, their cell densities approach wild-type levels by 48 hpf. In *myoVb* morphants, cell density increases over time and is significantly higher than that in wild-type. Scale bar = 50 μm. Asterisks indicate statistically significant difference between the wild type and the given genetic condition at a comparable time-point by Kruskal Wallis one-way ANOVA followed by Dunn’s post-hoc test. Quantification of cell height (F) of peridermal cells at 48 hpf in four genetic conditions mentioned. *grhl3* morphants and *grhl3;myoVb* double morphants show significantly reduced cell heights compared to those in control morphants and *myoVb* morphants, respectively (Kruskal Wallis one-way ANOVA, p<0.05). Orthogonal projections of confocal-scans showing cross-sectional view of periderm (G, H, I, J). Confocal micrographs of embryonic epidermis immunostained for Lgl/BrdU (K-R) at 18hpf (K-N) and 48hpf (O-R). Quantification of cell proliferation in the periderm at 18hpf, 4hpf, 30hpf,36hpf, and 48hpf in four genetic conditions (S). (All data presented as box plot showing median and IQR, One-way ANOVA with Tukey’s test, p < 0.05). In E, F and S square brackets denote comparisons while asterisks indicate statistically significant difference. Only relevant comparisons are shown. For all one-to-one comparisons, please refer to S2 Data.

Confocal micrographs indicated that *grhl3*;*myoVb* double morphants exhibited presence of larger peridermal cells than those in control and *myoVb* morphants (Fig 6A–6D). Further, quantification of cell height revealed that in *grhl3* morphant as well as in *grhl3*;*myoVb* double morphant embryos, peridermal cells were significantly flatter than those in control and *myoVb* morphants (Fig 6F). This trend was also reflected in the thickness of the periderm (Fig 6G–6J). To investigate the effect on cell-packing density in the absence of *grhl3* function, we imaged the dorsal head epidermis at a lower magnification in the *cldnb*:*lynEGFP* transgenic line. The peridermal cell packing density (cell number/mm^2^) estimated from such images at various developmental stages revealed that *myoVb* deficient embryos exhibit higher packing density as compared to wild type at 32, 36, and 48 hpf. However, both Grhl3 deficient and Grhl3;MyoVb double deficient embryos showed significantly lower packing density as compared to the wild type embryos at comparable time-points. Interestingly, both these conditions showed recovering trend in cell-packing density (Fig 6E, Kruskal Wallis one-way ANOVA with Dunn’s post hoc test). Despite this gradual recovery, there was profound heterogeneity in peridermal cell sizes in the *grhl3*;*myoVb* double morphant suggesting localized differences in the cell-densities (S3F Fig).

We further analyzed peridermal cell proliferation to check whether it compensates for the cell extrusion and to restore the cell density in *grhl3*MO and *grhl3;myoVb* MO embryos. We performed BrdU assay at 18hpf, 24hpf, 30hpf, 36hpf, and 48hpf (Fig 6K–6S). This analysis revealed that peridermal proliferation was higher in Grhl3 and MyoVb deficient embryos as compared to that in control embryos starting from 30hpf. Interestingly, peridermal proliferation in the double deficient embryos was higher than control embryos from 24hpf onwards and showed higher trend than Grhl3 deficient embryos with significant difference at 24, 36, and 48hpf (One way ANOVA with Tukey’s multiple comparison test, p < 0.05). These trends explain why the overall cell-packing density increases in a similar manner in both Grhl3 deficient and Grhl3;MyoVb double deficient embryos despite the extrusion being higher in latter.

To conclude, the epidermal architecture is altered in the absence of *grhl3* function. The peridermal cell packing density is lower in *grhl3* morphants as well as in *grhl3;myoVb* double morphants, especially at early stages, plausibly due to cell-extrusion. Besides, the epithelial cells are also flatter in these to genetic conditions. Increase in cell proliferation seems to aid in restoration of the packing density as the development proceeds.

### Grhl3 promotes the retention of peridermal cells by regulating levels of membrane E-cadherin

Loss of *grhl3* function in *myoVb* deficient embryos resulted in the altered epidermal architecture with localized differences in the cell-densities due to excessive cell extrusion. As discussed earlier, the transcriptome analysis followed by WISH showed a topical upregulation of adhesion molecules in the regions showing cell rounding. As the lateral adhesion may influence the process of cell retention, we asked whether *grhl3* functions via regulating levels of E-cadherin.

**Fig 7:**
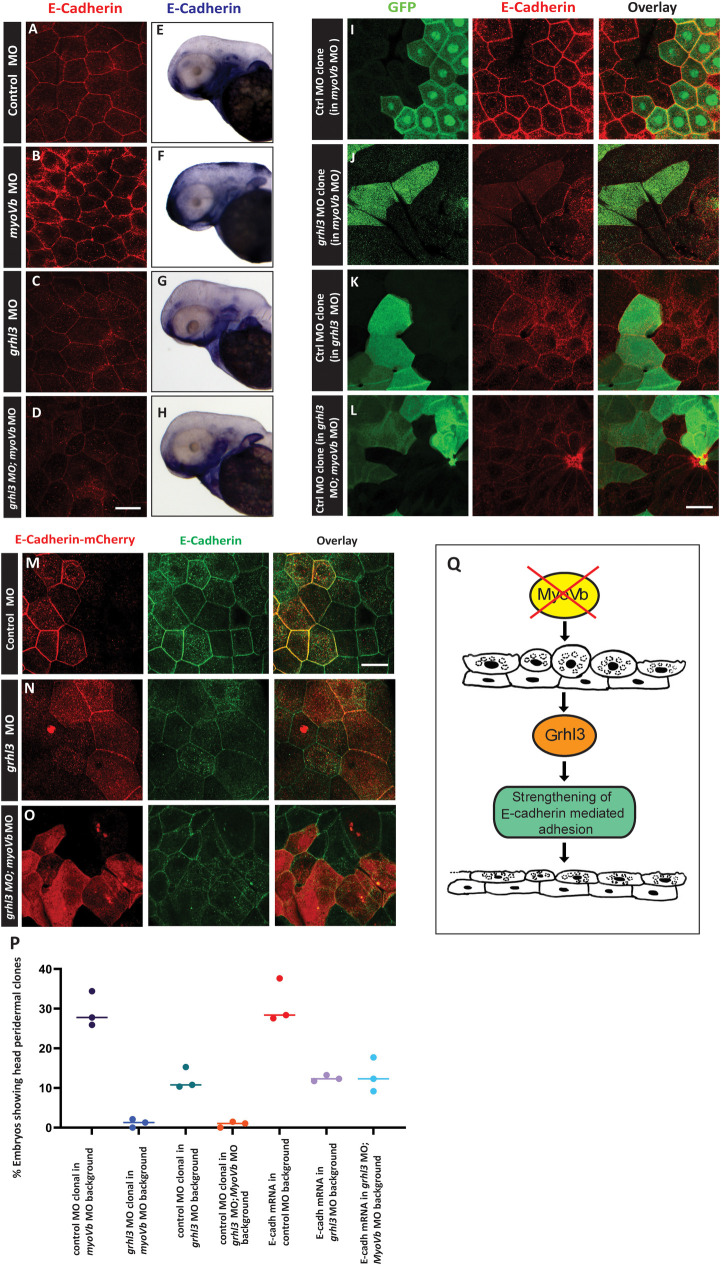
E-cadherin expression in MyoVb deficient epidermis is *grhl3*-dependent and cell autonomously sufficient for cell retention. Representative confocal images of dorsal head periderm of control (A), *myoVb* MO (B), *grhl3* MO (C), *grhl3* MO*;myoVb* MO (D) at 48 hpf, stained with anti-E-cadherin antibody (red). Scale bar in D (for A-D) = 50 μm. Increased localization of E-cadherin in *myoVb* morphants is absent in *grhl3;myoVb* double morphants. Representative bright field images showing expression of *cdh1* (E*-*cadherin) by WISH in the above four conditions (E-H). Note the increased patchy expression of *cdh1* adjacent to regions having cell rounding in *myoVb* morphants, which is absent in *grhl3;myoVb* double morphants. Assessment of retention probability of Grhl3 deficient cells in MyoVb deficient periderm. Control (I) and *grhl3* morphant clones (J) in *myoVb* morphant embryonic epidermis (I, J), control morphant clones (K,L) in *grhl3* morphant (K) and *grhl3;myoVb* double morphant epidermis (L). Clones are marked with GFP (green). Anti-E-cadherin staining marks cell boundaries (red). Confocal images of peridermal clones overexpressing Cdh1-mCherry (red) in control (M), *grhl3* morphant (N) and *grhl3;MyoVb* double morphant (O) epidermis. All cell boundaries are marked by anti-E-cadherin antibody (green). Scale bar in L, M = 50 μm. Graph representing proportion of embryos retaining the clones under given genetic conditions (P). % embryos showing peridermal clones per set are plotted as individual points, horizontal line denotes the median value. Note that *grhl3* morphant clones are retained rarely in *myoVb* morphant epidermis. While clones retention is very low in *grhl3*;*myoVb* double morphant embryos, *cdh1* overexpression partially rescues this phenotype. A schematic (Q) showing Grhl3 functioning in retention of rounded cells by strengthening E-cadherin mediated cell-cell adhesion.

Immunostainings with anti-E-Cadherin antibody revealed that despite the patchiness, membrane levels of E-Cadherin increase in *myoVb* morphant peridermal cells compared with those in control at 48 hpf (Fig 7A and 7B). The patchiness in E-cadherin localization is presumably a consequence of cell-cell rearrangements that take place at this stage, requiring junctional remodeling. However, combined loss of *grhl3* and *myoVb* resulted in much weaker E-Cadherin localization than that in *myoVb* or *grhl3* morphants (Fig 7C and 7D). Similarly, WISH analysis revealed that expression of *e-cadherin* decreases in MyoVb deficient embryos in the absence of *grhl3* function (Fig 7E–7H). These data suggest that the E-Cadherin localization in *myoVb* morphants, at 48 hpf is *grhl3* dependent. In the absence of *grhl3* function, the *myoVb* deficient peridermal cells fail to augment E-Cadherin levels.

To test the importance of *grhl3* function in MyoVb deficient embryos, we asked whether Grhl3 deficient clones are differentially retained in control versus *myoVb* morphant embryos. To inspect this facet, we generated *grhl3* MO clones in *myoVb* morphant embryos, wherein all peridermal cells were MyoVb deficient and the clone was additionally deficient for Grhl3. Estimation of clone retention revealed that *grhl3* MO clones had a near zero probability of being retained in the head periderm of MyoVb deficient embryos (Fig 7I–7L and 7P). Corroborating this result, we also found that in Grhl3;MyoVb double deficient embryos, control morphant clones were retained with very low frequency. These results, in addition to the preceding analysis (Fig 5G) wherein we did not see a difference between the retention of Grhl3 deficient and Grhl3;MyoVb double deficient clones in the wild-type background, indicate that in *myosin Vb* morphants junctional remodelling and cell-cell rearrangements (Sonal et al 2014) plausibly contribute towards additional shear stress and cell rounding up making the requirement of Grhl3 indispensable.

We used the Grhl3;MyoVb double deficient background to test whether E-Cadherin overexpression increased the retention probability of cells. Indeed, clonal overexpression of E-Cadherin significantly improved retention of clones in Grhl3;MyoVb double deficient embryos (Fig 7M–7P).

In summary, *grhl3* has an essential function in preventing cell extrusion. Moreover, this function of *grhl3* becomes more important in MyoVb deficient epidermis. Our data further suggest that E-cadherin cell autonomously promotes retention of cells in the combined absence of *grhl3* and *myoVb* functions.

## Discussion

Cells round up during cell division, cell-crowding and in various pathological conditions [37–42]. It is not clear whether there are mechanisms to retain and reintegrate rounded cells to maintain tissue homeostasis. Here, using the *gsp/myoVb* mutant, we have unraveled a Grhl3-E-cadherin dependent mechanism essential for retention of rounded up epidermal cells.

Our analyses indicate that cells rounding up due to perturbed membrane homeostasis are actively retained and reintegrated in the epidermis. *Grhl3*, a member of the Grainyhead family of transcription factors, is expressed specifically in the rounded up cells and plays an essential role in this process. MyoVb deficient peridermal cells exhibit higher levels of membrane E-cadherin, indicating increased cell-cell adhesion. This increased E-cadherin localization is not uniform, presumably due to junctional remodeling and cellular rearrangements in the periderm [45]. Our data indicate that the increased E-cadherin expression and localization in MyoVb deficient peridermal cells is induced by *grhl3* function. E-cadherin is sufficient for the retention of Grhl3;MyoVb double deficient clones in *gsp/myoVb* morphant embryos. Thus, *Grhl3* –E-cadherin axis plays an important role in retaining peridermal cells (Fig 7Q). Indeed, it has been shown that transcription factors belonging to Grainyhead family directly or indirectly control the expression of various adhesion components, including E-Cadherin [17, 18, 54, 55]. Interestingly, Grhl3 has also been shown to activate E-cadherin expression via an intronic enhancer [56]. Whether Grhl3 controls E-cadherin levels directly in the MyoVb deficient periderm remains to be elucidated.

Does retention of rounded cells contribute towards maintenance of epidermal homeostasis and architecture? Our analyses indicate that rounded up peridermal cells in MyoVb deficient embryos extrude in the absence of *grhl3* function. Besides, *grhl3* also functions in cell retention during early embryogenesis before 24 hpf. While we have not explored this developmental phenotype in detail, the cell-packing density data show a clear decrease in peridermal cell density in *grhl3* morphants at 26 hpf, plausibly a consequence of the early developmental cell extrusion prior to 24 hpf. The extrusion events–based on rosette analysis and cell extrusion analysis—are minimal in *grhl3* morphants after 24 hpf. A similar decrease in the cell-packing density is observed in *grhl3;myoVb* double morphants at 26 hpf. Interestingly, the cell packing density recovers in Grhl3 as well as Grhl3;MyoVb double deficient embryos. It is likely that the increased proliferation in both these genetic conditions is compensatory in nature and facilitates this recovery. Interestingly, however, in the double deficient embryos, there is a wave of peridermal cell extrusions post 32 hpf even as the cell density phenotype continues to recover. This wave of extrusions in double deficient embryos coincides with the occurrence of cell rounding up phenotype in MyoVb deficient embryos. The absence of cell rounding phenotype and abundant rosette formations indicate that rounded up cells might extrude rapidly rather than being retained. The time-lapse study corroborates this finding. Our analyses presented here suggest that *grhl3*, expressed in the rounded-up cells, is essential to facilitate their retention by up-regulating E-cadherin levels. While our data indicate that Grhl3 promotes adhesion and facilitate retention of rounded-up cells, it is not clear whether Grhl3 mediated adhesion also facilitates reintegration. In addition to this late function, it is possible that the early developmental function of *grhl3—*essential for the maintenance of early cell-packing density and thus presumably the tissue tension—also contributes towards the cell-retention in MyoVb deficient embryos. Further analysis using temporal inactivation will be required to tease apart the extent of early and late contribution of *grhl3* function in cell retention in the *myosin Vb* deficient background.

In the MyoVb deficient embryos, the cell packing density increases, which is consistent with the earlier observations that peridermal cells shrink in the absence of the motor function due to perturbed plasma membrane homeostasis and the increased cell-number compensates for the decrease in cell surface area [45]. Our cell height analysis reported here further indicates that peridermal cells are taller in the MyoVb deficient embryos. While in *grhl3* morphants the peridermal cells are shorter than those in the wild-type, they are even more squamous and constitute a peridermal layer that is much thinner in the double deficient embryos. In addition to cell proliferation, this increased squamation–as the peridermal cells are not being able to grow in the absence of *myoVb* function–possibly allows the tissue to compensate for the cell loss due to continued extrusion, albeit at the expense of the tissue getting thinner. Interestingly, despite significant restoration in packing density, the *grhl3;myoVb* double deficient embryos exhibit cell-size heterogeneity (S3F Fig), yielding patches of large but thin cells in the periderm, which along with reduced E-cadherin mediated adhesion, might form weak spots that potentially result in tissue tears and possibly the subsequent death of embryos.

Although termed as ‘master regulators of sealing and healing’ for their highly conserved role in epithelial homeostasis and repair [11], Grainyhead family transcription factors remain enigmatic, due to their diverse tissue specific functions as well as the functional redundancy between *grainyheadlike* paralogs. GRHL1 and GRHL2 have been reported to control cell differentiation and expression of various adhesion molecules in various epithelia to ensure proper morphogenesis, whereas GRHL3 is shown to drive peridermal differentiation and suppression of immune mediated hyperproliferation in the epidermis [22, 25]. Recently, cytoplasmic function of *Grhl3* has been shown to be important for acquiring mechanical properties that are essential for morphogenesis [16]. Our analyses show that *grhl3* function is also essential in epithelial cell retention in a normal developing periderm. This indicates that *grhl3* function might play a role in reintegration of cells undergoing mitotic rounding-up or in suppression of local crowding-induced homeostatic cell extrusion during epidermal morphogenesis; however, further investigation is necessary to explore these possibilities. Nevertheless, our analyses clearly show that *grhl3* function is essential to retain cells in a stressed epithelium like *myoVb* morphant periderm that has a higher propensity of cell-rounding up. Incidentally, *grhl3* is specifically up-regulated in psoriatic skin lesions and is essential for their healing [25]. Given the complex and multifaceted functions of *grhl3*, it would be interesting to test whether *Grhl3* acts in a manner similar to what we have shown, to facilitate epidermal repair in skin diseases.

## Materials and methods

### Ethics statement

Zebrafish rearing, handling and experimentation were approved by Institutional Animal Ethics committees at TIFR and La Trobe vide sanctions TIFR/IAEC/2013-3, TIFR/IAEC/2017-11, and La Trobe AEC16-91.

### Fish strains

The wild-type fish strains Tübingen (Tü) and DBS (wild-type strain derived from a local pet shop), and transgenic line Tg(cldnB:lynEGFP) [57] were used for knockdown analyses, live confocal analysis, cell surface area measurements. The *goosepimples/myoVb* [45] and *romeharsha* [46] mutant lines were used for monitoring the progression of balling up and transcriptome analysis. In situ hybridization analysis was done using albino strain.

### Generation of *grhl3*(-31) mutant via CRISPR/Cas9 mediated deletion

The target site for generation of *grhl3* single guide (sg) RNA was predicted using ZiFit (http://zifit.partners.org/ZiFiT/). Oligonucleotides (IDT) encoding the determined target site (TCGCAGAAATCATGGCAATC Reverse strand) were cloned into the plasmid pDR274, and sequence-verified plasmids were used as a template to transcribe sgRNA using the MEGAscript T7 kit (Ambion) as per manufactures recommendations. sgRNA and Cas9 nuclease (NEB M0386S) were co-injected into the cell at single-cell stage WT Tü zebrafish embryos. sgRNA was injected at concentrations ranging between 20–750 pg/nl, with greater efficiency of cutting observed at higher concentrations, as previously reported for other sgRNA [58, 59], and Cas9 protein was injected at a final concentration of 320 ng/μl.

To detect genomic lesions, DNA was isolated from embryos obtained from outcrossed injected fish in 20 μl 50 mM NaOH, and heated to 95°C for 10 min. The solution was neutralized by adding 2 μl 1 M Tris-HCl, pH 8.0. Samples were pulse centrifuged to pellet debris, and supernatant was used as a template for subsequent PCR reactions. PCR amplification of the regions flanking the target site, and subsequent sequencing using primers flanking the target site (grhl3_e4_SF 5’-CAGCTCTTCCCCTGAACTTG-3’, grhl3_e4_sR 5’-ACATAAATGCGGACCTCAGGTGT-3’) were used to determine genomic changes.

### Genotyping *grhl3* mutant embryos

During the assessment of genetic interaction between *myoVb* and *grhl3*, genomic DNA from single 5d old larva was extracted as above after the morphological evaluation of the cell rounding phenotype. PCR amplification was performed using the above primers, and 10 μl was run on a 3% agarose gel. The following band sizes were used to identify WT and *grhl3* mutant embryos; WT: 234 bp, *grhl3* (-31bp): 203 bp. In addition to or instead of genotyping, early phenotype of delayed epiboly was also used to identify *grhl3* mutant embryos.

### Comparative transcriptomics analysis using RNAseq

Fifty representative embryos each for *goosepimples* and *romeharsha* mutants, as well as respective siblings were fixed in 2 ml RNAlater (Thermo Fischer Scientific). The fixed embryos were processed by ILife Discoveries (Gurgaon, India), where mRNA was extracted using RNAlater protocol, and cDNA library (300 bp fragments) was prepared, followed by sequencing on Illumina GA 2000 with a predicted transcriptome coverage of 10× with 100 bp paired end reads. Raw data (~30 Gbp per sample) was filtered for read quality and reads were trimmed, using FastQC and FastX toolkit. Alignment was performed using TopHat 2.0 and RPKM values were calculated using DEGseq.

For comparative analysis of transcriptomes of *gsp* and *rhs*, transcripts with very low expression levels (RPKM<100), and transcripts showing significant difference (1.48 fold; 1.5 σ) in RPKM values between the two sibling samples were filtered out, to obtain 13,633 transcripts, with 1962 and 3532 differentially regulated transcripts in *gsp* and *rhs* mutants respectively. To further refine the data, we filtered out genes that did not show similar extent of misregulation between *gsp* and *rhs*. Further, transcripts showing dissimilar trends of regulation were filtered out by discarding those showing a difference in RPKM values by > 2-fold between the two mutants. As a result, we obtained 1349 (*gsp*) and 2605 (*rhs*) differentially regulated transcripts.

Over representation analysis and GO term for the common significantly regulated transcripts was performed using DAVID.

### Morpholino injections

In experiments concerning knockdown of *myoVb* function, previously published *myoVb* splice morpholino (*myoVb* MO- GATCTTCTATTACTGACCGAGTTGA) and corresponding five-base mismatch control morpholino (GAT**G**TT**G**TATTACT**C**ACC**C**A**C**TTGA) were used [45]. For knocking down *grhl3*, we used a morpholino targeted against the translation initiation site (*grhl3* MO- TGGTCATGACTCCTTGTACAGGCAG) and a four-base mismatch control (*grhl3 ctrl* MO- TGG**A**CAT**C**ACTCCT**G**GTACAGG**T**AG. The findings obtained using this morpholino were verified using three previously published morpholinos; two targeting splice junctions: exon 1-intron 1 (*grhl3* splice 1I1E- TAATCATAACACTTACTCAATCTCC) and exon4-intron4 (*grhl3* splice 4I4E-TGTATTACATAAATGCGGACCTCAG) and one targeting the translation start site (*grhl3* ATG2-TGAGAGCCTCAATCTCCTTGGTCAT) [28, 50]. All morpholinos were procured from Gene Tools, LLC (Eugene, Oregon, USA). For confocal analysis as well as clonal knockdown analysis, the grhl3 ATG morpholino was used.

Antisense morpholino oligos against *grhl3* and *myoVb*, as well as the respective control morpholinos were injected at 200 μM concentration. Injections were done at 1–2 cell stage, unless specified otherwise. For double knockdown experiments, a 1:1 mixture of *myoVb* splice MO and *grhl3* MO (400 μM each) was prepared to obtain a resultant concentration of 200 μM. Similarly, a 1:1 mixture of respective control morpholinos was used. For in situ hybridization experiments, to maintain a highly precise morpholino dose across single and double morpholino injections, instead of injection of 1:1 morpholino mixture, serial injections with *myoVb* splice MO and *grhl3* MO were performed, using the same needle used for single knockdown injections in the same set. All injections in a single set were performed in tandem, in a single session.

### Clonal morpholino knockdown and e-cadherin overexpression

For clonal knockdown of *myoVb*, *grhl3*, and combinations thereof, we used a mixture of morpholino solution containing single or double morpholinos with appropriate resultant concentrations and a GFP mRNA to locate morphant clones. Injections were done in a randomly selected cell at 16 or 32 cell stage. The zebrafish *e-cadherin-mCherry* construct was a gift from Prof. Erez Raz (Universität Münster). The E-cadherin mcherry mRNA was synthesised using SP6 Transcription Kit (ThermoFisher–Invitrogen, AM1340), purified using P-30 Gel Columns (BIORAD, 732–6250) and injected at 16/32 cell stage to achieve clonal overexpression.

### Quantification of clone retention

Clonally injected embryos were fixed and embryos showing GFP positive clones (generally 80–100% of total embryos) were sorted on Zeiss Apotome. Any embryos showing GFP expression in the whole embryo were discarded. The embryos with GFP positive clones represent successful clonal injections. These embryos were then stained as mentioned below using anti-GFP and anti-E-cadherin antibodies. Post glycerol upgradation, heads of all embryos were dissected out and mounted. These were thoroughly screened for presence of peridermal clones in the dorsal head region, using Zeiss LSM 510 or Zeiss LSM exciter, 40× objective. Clone retention percentage was calculated as:

100 × (Number of embryos showing peridermal clones in the dorsal head region / Total number of embryos with GFP labeled clones)

### Immunostaining

Immunostainings were carried out according to previously published protocol with some modifications (Sonawane et al., 2009). Briefly, embryos or larvae at the appropriate stages were fixed by overnight incubation at 4°C in 4% paraformaldehyde (PFA) in PBS, followed by methanol up-gradation and storage at—20°C (for cell size and cell proliferation analysis). For E-cadherin antibody staining as well as analysis of clonal knockdowns, the methanol upgradation was skipped. Embryos/larvae downgraded from methanol and those in PFA were washed with PBS followed by PBT (0.8% Triton X-100 in PBS), incubated in 10% NGS for 3 h at room temperature and followed by incubation with appropriate dilution of antibodies in 1% NGS in PBT for 4 hours at room temperature. The antibodies and their dilutions were as follows—rabbit anti-GFP (1:200, TorreyPines Biolabs; TP401), rabbit anti-Lgl2 (1:400; [60]), rat monoclonal anti-BrdU (1:50, Acris Antibodies; SM1667P), mouse anti-E cadherin (1:200, BD Bio transductions; 610182), anti activated Caspase 3 (1:200, BD pharmingen; 559565). Upon incubation, embryos were washed with 0.8% PBT and then incubated with secondary antibodies for 4 h at room temperature. Subsequently, embryos were washed in PBT, post fixed in 4% PFA, followed by PBS washes to remove PFA, and upgradation and mounting in Glycerol. The secondary antibodies used include Alexa 488 conjugated anti-rabbit and anti-mouse antibodies (1:250), Cy3 and Cy5 conjugated anti-rabbit, anti-mouse and anti-rat antibodies (1:750), from Invitrogen.

### Whole mount in Situ Hybridization (WISH)

Specific regions from *cdh1*, *cldn7b*, *cldne*, *oclna*, *dsc2l*, and *grhl3* (2208–2635 bp, 338–1065 bp, 90–818 bp, 928–1646 bp, 2206–2928 bp and 2140–2659 bp, respectively) were amplified using reverse transcribed total RNA isolated from 48 hpf embryos as a template. Subsequently, they were cloned into pCR TOPO 2.1 or pCR TOPOII (Invitrogen). For RNA probe synthesis, PCR amplicons obtained using appropriate generic primer sites flanking the probe sequence in the vector (T7, Sp6, M13F, M13R) were used as templates. Probes were synthesized using either T7 or SP6 RNA polymerase (Roche DIG RNA Labelling Kit). In situ hybridizations were performed as described earlier with a few modifications (Schulte-Merker, 2002). The stained samples were imaged using Zeiss SteREO Discovery coupled with AxioCam.

### Image acquisition and processing

Imaging of immunostainings was done in the dorsal head epidermis, using either Zeiss LSM 510 Meta with Plan apochromat 639/1.40 oil or EC Plan-Neofluar 409/1.30 oil objective or Zeiss LSM 710/880 with Plan-apochromat 639/1.40 oil. Optical zoom of 63x was used for most of the images. The image acquisition was done with pinhole size of 1 AU, frame size of 1024 × 1024 pixels, with averaging of 4, and bit depth of 8 or 16. The PMT gains were adjusted to capture maximum possible dynamic range.

For live confocal imaging, larvae were embedded in 0.2% Low melting agarose (Sigma-Aldrich), either placed in petri dishes having glass coverslips at the bottom and imaged on LSM5 exciter, LSM 710 or LSM 880 with 40X Plan-apochromat objective/1.4NA with 1X/2X Zoom. For longer imaging experiments to capture cell extrusions, embryos were partially submerged in agarose, exposing the dorsal side of their head. E3 was added on top to cover the entire surface of the plate to avoid dehydration. Water was carefully replenished without disturbing the frame every 30 min. Zeiss 880 with 40x water objective was used for scanning. Bright field imaging was done using embryos mounted in methylcellulose (3%), either on Zeiss SteREO Discovery with AxioCam or Nikon SMZ18 with a Nikon DS-Ri2 CMOS camera. ImageJ (FIJI) was used for image processing and analysis.

Cell circularities were measured using the ‘shape descriptor’ measurement in imageJ. Circularity = 4π(area/perimeter)^2^

### BrdU incorporation assay and analysis of proliferation

Embryos were dechorionated at 18hpf, 24hpf, 30hpf, 36hpf, and 48 hpf, and incubated in 10 mM solution of BrdU in 2% DMSO in E3 for 3h at 29°C. After incubation, followed by 5 min washes with E3 buffer, embryos were fixed in 4% PFA and upgraded in methanol. After downgrading to PBS and prior to immunostaining, the fixed larvae were treated with 4N HCl for 20 min at room temperature.

Quantification of proliferation in the periderm and basal epidermis was done using confocal stacks. Nuclei positive for BrdU staining, as well as total cells (based on Lgl staining demarcating cell boundaries) were counted using cell counter plugin in ImageJ. Percentage of cells positive for BrdU out of total cells was used as a proliferation index in the epidermis. Five animals per set per genetic condition and two such experimental sets were analyzed for proliferation. One-way ANOVA followed by Tukey’s test was used for determining statistically significant differences between groups.

### Cell height quantification

The cell heights were estimated by counting the number of stacks showing lynEGFP staining for individual cells. Height of five cells were measured from each embryo, total 15 embryos from three sets were used for the analysis. Kruskal-Wallis one-way ANOVA with Dunn’s test was used for determining significant differences between groups.

### Cell density and rosette analysis

Tg(cldnB:lynEGFP) embryos injected with either *myoVb* MO, *grhl3* MO or a combination of both and were fixed and stained with anti GFP antibody. Images were acquired as described above using 40X oil objective at 0.6X zoom. Number of cells and total area occupied by them were calculated using ImageJ. Cell density was calculated as cells /mm^2^. Similarly, number of cell-rosettes were counted and expressed as rosettes/mm^2^. Between 35–60 embryos per genetic condition per stage were analyzed from three independent experiments. Kruskal-Wallis one-way ANOVA followed by Dunn’s test was used for determining statistically significant differences between groups.

### Cell extrusion assay

Wild-type embryos were injected with *myoVb* MO, *grhl3* MO or a combination of both and used for quantification of cell extrusion occurring between 12–18 hpf, 18–24 hpf, 24–30 hpf, 30–36 hpf, and 36–42 hpf. Twenty (or ten for 36 hpf-43 hpf) embryos were dechorionated and transferred to Eppendorf tubes containing 2 ml E3 buffer and incubated at 29° C for 6 h. The E3 buffer used for incubation was collected and embryos were washed thrice with 2 ml E3 buffer; the buffer used for washing was pooled with the buffer used for incubation. This was then centrifuged for 5 min at 1000×g and supernatant was carefully discarded. The pellet was re-suspended in 10μL of 0.1% Trypan blue solution (Sigma Aldrich, T6146) and incubated at 29° C for 10 min. the solution was well mixed and mounted on a slide and dead cells were counted manually under a light microscope using a 20X objective (Zeiss LSM 880 or Zeiss Apotome). Five replicates were performed. Statistically significant differences between groups were determined using one-way ANOVA followed by Tukey’s test.

### Statistical analysis

GraphPad PRISM and Microsoft Excel (2019) were used for statistical analysis and plotting graphs. Along with this, an online tool http://shiny.chemgrid.org/boxplotr/ was used to create box plots. Details of all the statistical tests and comparisons are presented in S2 Data.

## Supporting information

S1 Movie Live temporally cropped confocal movie of *myoVb* morphant head periderm showing regression of cell rounding from just before 50 hpf and ending at 55.5 hpf (Maximum intensity projection, acquisition: 53 seconds/stack, playback: 8 frames/sec). Cell boundaries as well as intracellular membranous compartments are marked by lyn-EGFP. During the recovery period, rounded cells progressively become polygonal, however accumulation of intracellular vesicles persists.
(AVI)

S2 Movie Live temporally and frame-wise cropped confocal movie of *grhl3;myoVb* morphant head periderm between 36–38 hpf showing cell extrusions (red arrows). Neighboring cells round up and get extruded in a cascade effect. (Maximum intensity projection, acquisition: 97 seconds/stack, playback: 5 frames/sec).
(AVI)

S3 Movie Live temporally and frame-wise cropped confocal movie of *grhl3;myoVb* morphant head periderm between 36–38 hpf showing cell extrusions (red arrows). Neighboring cells round up and get extruded in a cascade effect. (Maximum intensity projection, acquisition: 97 seconds/stack, playback: 5 frames/sec).
(AVI)

S4 Movie Live temporally and frame-wise cropped confocal movie of *myoVb* morphant head periderm between 36–38 hpf showing cell extrusion (red arrow). Similar to the above movie, neighboring cells are seen rounding up, however, they do not get extruded, but form a cluster of rounded cells. (Maximum intensity projection, acquisition: 67 seconds/stack, playback: 5 frames/sec)
(AVI)

S5 Movie Live temporally and frame-wise cropped confocal movie of *grhl3* morphant head periderm between 36–38 hpf. No extrusions were visible (Maximum intensity projection, acquisition: 97 seconds/stack, playback: 3 frames/sec)
(AVI)

S6 Movie Live temporally and frame-wise cropped confocal movie of control morphant head periderm between 36–38 hpf. Peridermal cells do not show extensive basolateral projections. (Maximum intensity projection, acquisition: 90 seconds/stack, playback: 4 frames/sec)
(AVI)

S7 Movie Live temporally and frame-wise cropped confocal movie of *grhl3* morphant head periderm between 36–38 hpf. Peridermal cells show basolateral projections that actively move (red arrow). This suggests decreased cadherin dependent basolateral adhesion (Maximum intensity projection, acquisition: 90 seconds/stack, playback: 4 frames/sec)
(AVI)

S8 Movie Live temporally and frame-wise cropped confocal movie of *grhl3;myoVb* double morphant head periderm between 36–38 hpf. Peridermal cells show abundant and highly motile basolateral projections (red arrow). This suggests extremely weak cadherin dependent basolateral adhesion (Maximum intensity projection, acquisition: 90 seconds/stack, playback: 4 frames/sec)
(AVI)

S1 Fig Percent embryos (A) showing accumulation of cell debris inside chorion and survival at 72 hpf upon injection of various morpholinos. Control and *grhl3* morphant embryos showing mild deformation of the finfold at 36 hpf (B). DIC images of *gsp/*myoVb mutant injected with *grhl3* MO (C). Note the absence of cell rounding in *grhl3* MO injected *gsp/*myoVb mutant while the uninjected mutant shows rounded cells. Generation of *grhl3* (-31) mutant (D). Exonic structure of *grlh3* mRNA with exons alternatively colored white and grey. Red arrowhead indicates the location of guide-RNA target site in exon 4. Grhl3 protein domain structure, with transactivation domain shown in orange, DNA binding domain in blue, and the dimerization domain in yellow. Dashed lines indicate start and end of coding region. Deletion of 31bp causes a frame shift after amino acid 143, resulting in a truncated protein with 81 missense amino acids that lacks the DNA binding domain and dimerization domain. These missense amino acids are indicated in red in the schematic. Exon sizes are shown to scale relative to the size of the coding region (Modified from Miles et al., 2017). Sequence of exon 4 region of *grhl3*, showing the deletion of 31bp. GuideRNA sequence (reverse orientation) is indicated in orange while blue indicates PAM sequence. Deleted bases are indicated by red dashes. E. DIC images of *grhl3(-31/-31)* sibling and mutant at 5 dpf. Note the defective lower law in the mutant (red arrowhead). F. Quantification of cell-debris accumulation in the chorion in *grhl3 (-31/-31)*.
(TIF)

S2 Fig Sense controls for Whole mount In-Situ Hybridization (WISH). Sense controls for *grhl3* in control (A) and *myoVb* MO embryos (B) at the given developmental time points.
(TIF)

S3 Fig Representative stereomicroscopy images (A) of control morphants, *myoVb* morphants, *grhl3* morphants, and *myoVb;grhl3* double morphants at various time points showing progressive accumulation of cell debris (black arrowheads) in the chorion. Quantification of cell shedding using Trypan blue staining for control morphants, *grhl3* morphants, *myoVb* morphants, and *grhl3;myoVb* morphants between 36 hpf and 42 hpf (B). Asterisks indicate statistically significant difference by One-way ANOVA with Tukey’s test at p<0.05. For all statistical comparisons please refer to S2 Data Representative time-lapse DIC images (C) of *grhl3* MO*; myoVb* MO embryo mounted in E3 buffer. Colored arrowheads indicate the same extruding cells at different time-points. Representative live confocal images (D) of cldnB:lynEGFP embryos injected with control MO, *grhl3* MO, *myoVb* MO, and *grhl3* MO*; myoVb* MO and digitally zoomed versions (D’) of the boxed regions in D. Yellow triangles indicate profuse basolateral projections in the absence of *grhl3* function, indicating weakened cell adhesion. Scale bar = 50 μm. Confocal images along with orthogonal projections of apoptotic cells, undergoing apical extrusion or delamination (E). Apoptotic cells were rarely observed. Activated caspase 3 is in Red while lynEGFP is in green. Scale bar = 20 μm. Maximum intensity projections of head periderm at 48 hpf of wild-type, *grhl3* morphants, *myoVb* morphants, and *grhl3;myoVb* double morphants (F). *Grhl3;myoVb* morphant periderm shows a heterogeneity with patches of large and small cells. White asterisks in F indicate patches of big peridermal cells.
(TIF)

S4 Fig A plot of cell circularity values over time for 30 randomly selected cells (black traces) from four movies in wildtype (A), *grhl3* MO (B), *myoVb* MO (C), and *grhl3;myoVb* MO (D) starting at 50hpf. Cell circularity of four extruding cells (red traces) in *myoVb* MO (C) and *grhl3;myoVb* MO (D) chosen from four movies are also shown. Green dotted line represents cell circularity threshold used for classifying rounded cells. As expected, extruding cells attain high circularity values prior to extrusion. Table E showing analysis of total number of cells and number of rounded cells, apically extruded cells and delaminated (basal cell extrusion) cells in *grhl3;myoVb* MO during 50 to 53 hpf. The duration of the movie is indicated in parentheses.
(TIF)

S1 Data Transcriptomic analysis of *gsp* and *rhs* mutants and lists of common up and down-regulated genes. (XLSX)

S2 Data Figure-wise numerical data and statistical analysis. (XLSX)

## References

[1] Sumigray KD, Lechler T. Cell adhesion in epidermal development and barrier formation. Current Topics in Developmental Biology. 2015. doi: 10.1016/bs.ctdb.2014.11.027 25733147 PMC4737682

[2] Morita K, Miyachi Y, Furuse M. Tight junctions in epidermis: From barrier to keratinization. European Journal of Dermatology. 2011. doi: 10.1684/ejd.2010.1192 21300606

[3] Sugawara T, Iwamoto N, Akashi M, Kojima T, Hisatsune J, Sugai M, et al. Tight junction dysfunction in the stratum granulosum leads to aberrant stratum corneum barrier function in claudin-1-deficient mice. J Dermatol Sci. 2013. doi: 10.1016/j.jdermsci.2013.01.002 23433550

[4] Georges-Labouesse E, Messaddeq N, Yehia G, Cadalbert L, Dierich A, Le Meur M. Absence of integrin α6 leads to epidermolysis bullosa and neonatal death in mice. Nat Genet. 1996. doi: 10.1038/ng0796-370 8673141

[5] Tsuruta D, Hashimoto T, Hamill KJ, Jones JCR. Hemidesmosomes and focal contact proteins: Functions and cross-talk in keratinocytes, bullous diseases and wound healing. Journal of Dermatological Science. 2011. doi: 10.1016/j.jdermsci.2011.01.005 21376539 PMC4492441

[6] Hamill KJ, Hiroyasu S, Colburn ZT, Ventrella R V., Hopkinson SB, Skalli O, et al. Alpha Actinin-1 Regulates Cell-Matrix Adhesion Organization in Keratinocytes: Consequences for Skin Cell Motility. J Invest Dermatol. 2015. doi: 10.1038/jid.2014.505 25431851 PMC4366307

[7] Walko G, Castañón MJ, Wiche G. Molecular architecture and function of the hemidesmosome. Cell and Tissue Research. 2015. doi: 10.1007/s00441-014-2061-z 26017636 PMC4452579

[8] M’Boneko V, Merker HJ. Development and morphology of the periderm of mouse embryos (days 9–12 of gestation). Acta Anat (Basel). 1988. doi: 10.1159/000146662 3227794

[9] Sonawane M, Carpio Y, Geisler R, Schwarz H, Maischein HM, Nuesslain-Volhard C. Zebrafish penner/lethal giant larvae 2 functions in hemidesmosome formation, maintenance of cellular morphology and growth regulation in the developing basal epidermis. Development. 2005. doi: 10.1242/dev.01904 15983403

[10] Mace KA, Pearson JC, McGinnis W. An epidermal barrier wound repair pathway in Drosophila is mediated by grainy head. Science (80-). 2005. doi: 10.1126/science.1107573 15831751

[11] Stramer B, Martin P. Cell biology: Master regulators of sealing and healing. Current Biology. 2005. doi: 10.1016/j.cub.2005.05.034 15936265

[12] Caddy J, Wilanowski T, Darido C, Dworkin S, Ting SB, Zhao Q, et al. Epidermal Wound Repair is Regulated by the Planar Cell Polarity Signaling Pathway. Dev Cell. 2010. doi: 10.1016/j.devcel.2010.06.008 20643356 PMC2965174

[13] Paré A, Kim M, Juarez MT, Brody S, McGinnis W. The functions of Grainy head-like proteins in animals and fungi and the evolution of apical extracellular barriers. PLoS One. 2012. doi: 10.1371/journal.pone.0036254 22590528 PMC3348937

[14] Cangkrama M, Darido C, Georgy SR, Partridge D, Auden A, Srivastava S, et al. Two Ancient Gene Families Are Critical for Maintenance of the Mammalian Skin Barrier in Postnatal Life. J Invest Dermatol. 2016. doi: 10.1016/j.jid.2016.02.806 26975724

[15] Cristo I, Carvalho L, Ponte S, Jacinto A. Novel role for Grainy head in the regulation of cytoskeletal and junctional dynamics during epithelial repair. J Cell Sci. 2018. doi: 10.1242/jcs.213595 30131442

[16] Kimura-Yoshida C, Mochida K, aki Nakaya M, Mizutani T, Matsuo I. Cytoplasmic localization of GRHL3 upon epidermal differentiation triggers cell shape change for epithelial morphogenesis. Nat Commun. 2018. doi: 10.1038/s41467-018-06171-8 30283008 PMC6170465

[17] Werth M, Walentin K, Aue A, Schönheit J, Wuebken A, Pode-Shakked N, et al. The transcription factor grainyhead-like 2 regulates the molecular composition of the epithelial apical junctional complex. Development. 2010. doi: 10.1242/dev.055483 20978075

[18] Varma S, Cao Y, Tagne JB, Lakshminarayanan M, Li J, Friedman TB, et al. The transcription factors grainyhead-like 2 and NK2-homeobox 1 form a regulatory loop that coordinates lung epithelial cell morphogenesis and differentiation. J Biol Chem. 2012. doi: 10.1074/jbc.M112.408401 22955271 PMC3481326

[19] Ting SB, Caddy J, Wilanowski T, Auden A, Cunningham JM, Elias PM, et al. The epidermis of Grhl3-null mice displays altered lipid processing and cellular hyperproliferation. Organogenesis. 2005. doi: 10.4161/org.2.2.2167 19521564 PMC2634083

[20] Yu Z, Bhandari A, Mannik J, Pham T, Xu X, Andersen B. Grainyhead-like factor Get1/Grhl3 regulates formation of the epidermal leading edge during eyelid closure. Dev Biol. 2008. doi: 10.1016/j.ydbio.2008.04.001 18485343 PMC2494567

[21] Yu Z, Mannik J, Soto A, Lin KK, Andersen B. The epidermal differentiation-associated Grainyhead gene Get1/Grhl3 also regulates urothelial differentiation. EMBO J. 2009. doi: 10.1038/emboj.2009.142 19494835 PMC2711180

[22] Hopkin AS, Gordon W, Klein RH, Espitia F, Daily K, Zeller M, et al. GRHL3/GET1 and trithorax group members collaborate to activate the epidermal progenitor differentiation program. PLoS Genet. 2012. doi: 10.1371/journal.pgen.1002829 22829784 PMC3400561

[23] Darido C, Georgy SR, Wilanowski T, Dworkin S, Auden A, Zhao Q, et al. Targeting of the Tumor Suppressor GRHL3 by a miR-21-Dependent Proto-Oncogenic Network Results in PTEN Loss and Tumorigenesis. Cancer Cell. 2011. doi: 10.1016/j.ccr.2011.10.014 22094257

[24] Bhandari A, Gordon W, Dizon D, Hopkin AS, Gordon E, Yu Z, et al. The Grainyhead transcription factor Grhl3/Get1 suppresses miR-21 expression and tumorigenesis in skin: Modulation of the miR-21 target MSH2 by RNA-binding protein DND1. Oncogene. 2013. doi: 10.1038/onc.2012.168 22614019 PMC4026359

[25] Gordon WM, Zeller MD, Klein RH, Swindell WR, Ho H, Espetia F, et al. A GRHL3-regulated repair pathway suppresses immune-mediated epidermal hyperplasia. J Clin Invest. 2014. doi: 10.1172/JCI77138 25347468 PMC4348962

[26] Goldie SJ, Cottle DL, Tan FH, Roslan S, Srivastava S, Brady R, et al. Loss of GRHL3 leads to TARC/CCL17-mediated keratinocyte proliferation in the epidermis. Cell Death Dis. 2018. doi: 10.1038/s41419-018-0901-6 30341279 PMC6195598

[27] Chalmers AD, Lachani K, Shin Y, Sherwood V, Cho KWY, Papalopulu N. Grainyhead-like 3, a transcription factor identified in a microarray screen, promotes the specification of the superficial layer of the embryonic epidermis. Mech Dev. 2006. doi: 10.1016/j.mod.2006.04.006 16916602

[28] De La Garza G, Schleiffarth JR, Dunnwald M, Mankad A, Weirather JL, Bonde G, et al. Interferon regulatory factor 6 promotes differentiation of the periderm by activating expression of grainyhead-like 3. J Invest Dermatol. 2013. doi: 10.1038/jid.2012.269 22931925 PMC3541433

[29] Peyrard-Janvid M, Leslie EJ, Kousa YA, Smith TL, Dunnwald M, Magnusson M, et al. Dominant mutations in GRHL3 cause Van der Woude syndrome and disrupt oral periderm development. Am J Hum Genet. 2014. doi: 10.1016/j.ajhg.2013.11.009 24360809 PMC3882735

[30] Miles LB, Darido C, Kaslin J, Heath JK, Jane SM, Dworkin S. Mis-expression of grainyhead-like transcription factors in zebrafish leads to defects in enveloping layer (EVL) integrity, cellular morphogenesis and axial extension. Sci Rep. 2017. doi: 10.1038/s41598-017-17898-7 29242584 PMC5730563

[31] Herrera-Perez RM, Kasza KE. Biophysical control of the cell rearrangements and cell shape changes that build epithelial tissues. Current Opinion in Genetics and Development. 2018. doi: 10.1016/j.gde.2018.07.005 30103186

[32] Gibson WT, Gibson MC. Chapter 4 Cell Topology, Geometry, and Morphogenesis in Proliferating Epithelia. Current Topics in Developmental Biology. 2009. doi: 10.1016/S0070-2153(09)89004-2 19737643

[33] Slanchev K, Carney TJ, Stemmler MP, Koschorz B, Amsterdam A, Schwarz H, et al. The epithelial cell adhesion molecule EpCAM is required for epithelial morphogenesis and integrity during zebrafish epiboly and skin development. PLoS Genet. 2009. doi: 10.1371/journal.pgen.1000563 19609345 PMC2700972

[34] Sampedro MF, Izaguirre MF, Sigot V. E-cadherin expression pattern during zebrafish embryonic epidermis development. F1000Research. 2018. doi: 10.12688/f1000research.15932.3 30473778 PMC6234749

[35] Kondo T, Hayashi S. Mitotic cell rounding accelerates epithelial invagination. Nature. 2013. doi: 10.1038/nature11792 23334416

[36] Hoijman E, Rubbini D, Colombelli J, Alsina B. Mitotic cell rounding and epithelial thinning regulate lumen growth and shape. Nat Commun. 2015. doi: 10.1038/ncomms8355 26077034

[37] Gibson WT, Veldhuis JH, Rubinstein B, Cartwright HN, Perrimon N, Brodland GW, et al. Control of the mitotic cleavage plane by local epithelial topology. Cell. 2011. doi: 10.1016/j.cell.2010.12.035 21295702 PMC3491649

[38] Lancaster O, LeBerre M, Dimitracopoulos A, Bonazzi D, Zlotek-Zlotkiewicz E, Picone R, et al. Mitotic Rounding Alters Cell Geometry to Ensure Efficient Bipolar Spindle Formation. Dev Cell. 2013. doi: 10.1016/j.devcel.2013.03.014 23623611

[39] Marinari E, Mehonic A, Curran S, Gale J, Duke T, Baum B. Live-cell delamination counterbalances epithelial growth to limit tissue overcrowding. Nature. 2012. doi: 10.1038/nature10984 22504180

[40] Kocgozlu L, Saw TB, Le AP, Yow I, Shagirov M, Wong E, et al. Epithelial Cell Packing Induces Distinct Modes of Cell Extrusions. Curr Biol. 2016. doi: 10.1016/j.cub.2016.08.057 27746027 PMC5423527

[41] Saw TB, Doostmohammadi A, Nier V, Kocgozlu L, Thampi S, Toyama Y, et al. Topological defects in epithelia govern cell death and extrusion. Nature. 2017. doi: 10.1038/nature21718 28406198 PMC5439518

[42] Gudipaty SA, Rosenblatt J. Epithelial cell extrusion: Pathways and pathologies. Seminars in Cell and Developmental Biology. 2017. doi: 10.1016/j.semcdb.2016.05.010 27212253 PMC5116298

[43] Franco JJ, Atieh Y, Bryan CD, Kwan KM, Eisenhoffer GT. Cellular crowding influences extrusion and proliferation to facilitate epithelial tissue repair. Mol Biol Cell. 2019. doi: 10.1091/mbc.E18-05-0295 30785842 PMC6727764

[44] Bergstralh DT, Lovegrove HE, St. Johnston D. Lateral adhesion drives reintegration of misplaced cells into epithelial monolayers. Nat Cell Biol. 2015. doi: 10.1038/ncb3248 26414404 PMC4878657

[45] Sonal, Sidhaye J, Phatak M, Banerjee S, Mulay A, Deshpande O, et al. Myosin Vb Mediated Plasma Membrane Homeostasis Regulates Peridermal Cell Size and Maintains Tissue Homeostasis in the Zebrafish Epidermis. PLoS Genet. 2014. doi: 10.1371/journal.pgen.1004614 25233349 PMC4169241

[46] Phatak M, Sonawane M. Functional characterisation of romeharsha and clint1 reaffirms the link between plasma membrane homeostasis, cell size maintenance and tissue homeostasis in developing zebrafish epidermis. J Biosci. 2018. doi: 10.1007/s12038-018-9777-y 30207308

[47] Van Eeden FJM, Granato M, Schach U, Brand M, Furutani-Seiki M, Haffter P, et al. Genetic analysis of fin formation in the zebrafish, Danio rerio. Development. 1996. 9007245 10.1242/dev.123.1.255

[48] Bonnet MC, Preukschat D, Welz PS, Van Loo G, Ermolaeva MA, Bloch W, et al. The Adaptor Protein FADD Protects Epidermal Keratinocytes from Necroptosis In Vivo and Prevents Skin Inflammation. Immunity. 2011. doi: 10.1016/j.immuni.2011.08.014 22000287

[49] Jänicke M, Renisch B, Hammerschmidt M. Zebrafish grainyhead-like1 is a common marker of different non-keratinocyte epidermal cell lineages, which segregate from each other in a Foxi3-dependent manner. Int J Dev Biol. 2010. doi: 10.1387/ijdb.092877mj 19757382 PMC3408584

[50] Dworkin S, Simkin J, Darido C, Partridge DD, Georgy SR, Caddy J, et al. Grainyhead-like 3 regulation of endothelin-1 in the pharyngeal endoderm is critical for growth and development of the craniofacial skeleton. Mech Dev. 2014. doi: 10.1016/j.mod.2014.05.005 24915580

[51] Kim S, Lewis AE, Singh V, Ma X, Adelstein R, Bush JO. Convergence and Extrusion Are Required for Normal Fusion of the Mammalian Secondary Palate. PLoS Biol. 2015. doi: 10.1371/journal.pbio.1002122 25848986 PMC4388528

[52] Ohsawa S, Vaughen J, Igaki T. Cell Extrusion: A Stress-Responsive Force for Good or Evil in Epithelial Homeostasis. Developmental Cell. 2018. doi: 10.1016/j.devcel.2018.01.009 29408235 PMC6207186

[53] Blankenship JT, Backovic ST, Sanny JSSP, Weitz O, Zallen JA. Multicellular Rosette Formation Links Planar Cell Polarity to Tissue Morphogenesis. Dev Cell. 2006. doi: 10.1016/j.devcel.2006.09.007 17011486

[54] Wilanowski T, Caddy J, Ting SB, Hislop NR, Cerruti L, Auden A, et al. Perturbed desmosomal cadherin expression in grainy head-like 1-null mice. EMBO J. 2008. doi: 10.1038/emboj.2008.24 18288204 PMC2274933

[55] Klein RH, Lin Z, Hopkin AS, Gordon W, Tsoi LC, Liang Y, et al. GRHL3 binding and enhancers rearrange as epidermal keratinocytes transition between functional states. PLoS Genet. 2017. doi: 10.1371/journal.pgen.1006745 28445475 PMC5425218

[56] Alotaibi H, Basilicata MF, Shehwana H, Kosowan T, Schreck I, Braeutigam C, et al. Enhancer cooperativity as a novel mechanism underlying the transcriptional regulation of E-cadherin during mesenchymal to epithelial transition. Biochim Biophys Acta—Gene Regul Mech. 2015. doi: 10.1016/j.bbagrm.2015.01.005 25652130

[57] Haas P, Gilmour D. Chemokine Signaling Mediates Self-Organizing Tissue Migration in the Zebrafish Lateral Line. Dev Cell. 2006. doi: 10.1016/j.devcel.2006.02.019 16678780

[58] Hwang WY, Fu Y, Reyon D, Maeder ML, Tsai SQ, Sander JD, et al. Efficient genome editing in zebrafish using a CRISPR-Cas system. Nat Biotechnol. 2013. doi: 10.1038/nbt.2501 23360964 PMC3686313

[59] Shah AN, Davey CF, Whitebirch AC, Miller AC, Moens CB. Rapid reverse genetic screening using CRISPR in zebrafish. Nat Methods. 2015. doi: 10.1038/nmeth.3360 25867848 PMC4667794

[60] Sonawane M, Martin-Maischein H, Schwarz H, Nusslein-Volhard C. Lgl2 and E-cadherin act antagonistically to regulate hemidesmosome formation during epidermal development in zebrafish. Development. 2009. doi: 10.1242/dev.032508 19261700

