# Supplementary figures and images for: Grhl3 promotes retention of epidermal cells under endocytic stress to maintain epidermal architecture in zebrafish
Source: PLoS Genet. 2021 Sep 27;17(9):e1009823. doi: 10.1371/journal.pgen.1009823 (PMC8496789; doi:10.1371/journal.pgen.1009823)

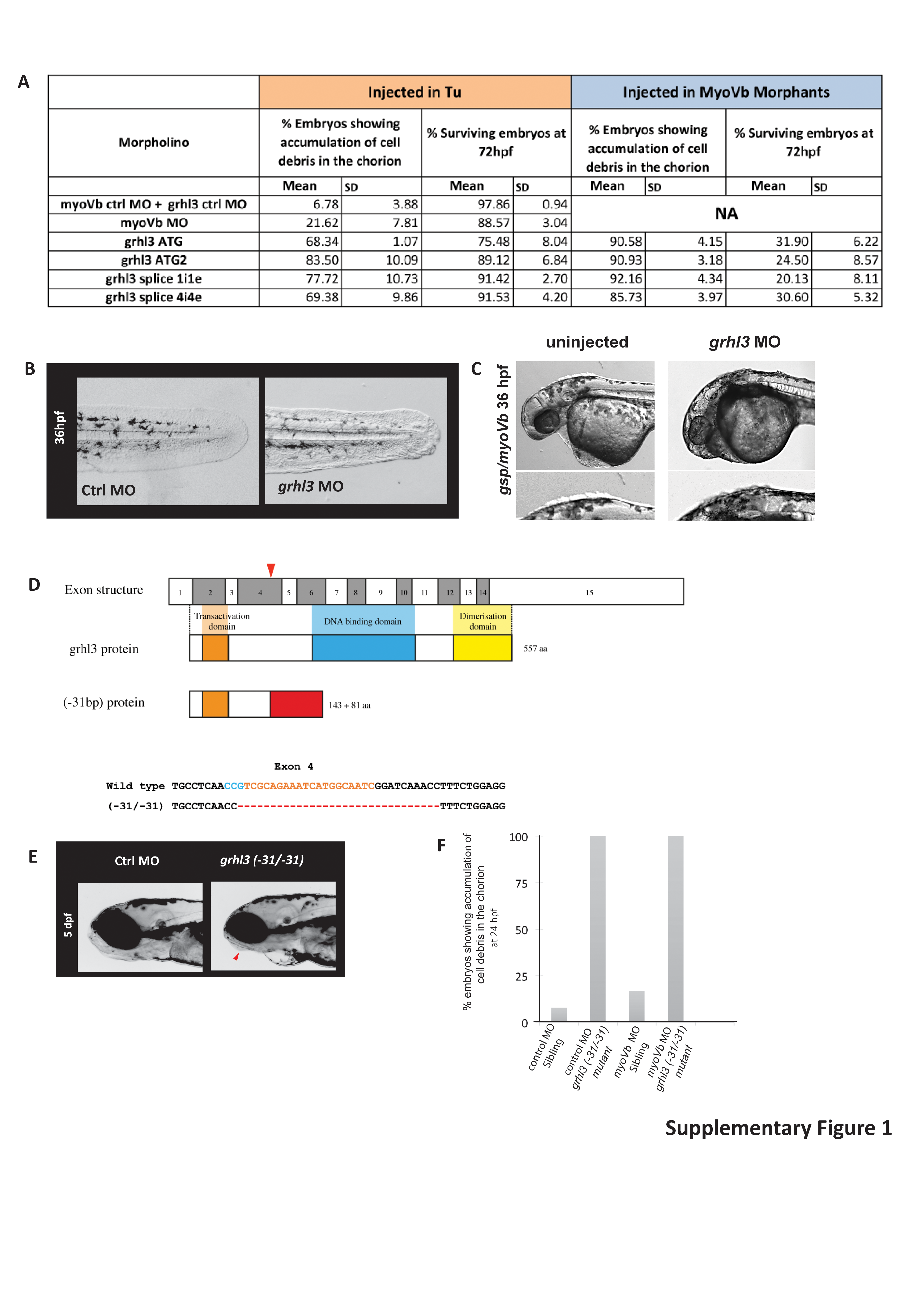

Supplement: S1 Fig — Percent embryos (A) showing accumulation of cell debris inside chorion and survival at 72 hpf upon injection of various morpholinos. Control and grhl3 morphant embryos showing mild deformation of the finfold at 36 hpf (B). DIC images of gsp/myoVb mutant injected with grhl3 MO (C). Note the absence of cell rounding in grhl3 MO injected gsp/myoVb mutant while the uninjected mutant shows rounded cells. Generation of grhl3 (-31) mutant (D). Exonic structure of grlh3 mRNA with exons alternatively colored white and grey. Red arrowhead indicates the location of guide-RNA target site in exon 4. Grhl3 protein domain structure, with transactivation domain shown in orange, DNA binding domain in blue, and the dimerization domain in yellow. Dashed lines indicate start and end of coding region. Deletion of 31bp causes a frame shift after amino acid 143, resulting in a truncated protein with 81 missense amino acids that lacks the DNA binding domain and dimerization domain. These missense amino acids are indicated in red in the schematic. Exon sizes are shown to scale relative to the size of the coding region (Modified from Miles et al., 2017). Sequence of exon 4 region of grhl3, showing the deletion of 31bp. GuideRNA sequence (reverse orientation) is indicated in orange while blue indicates PAM sequence. Deleted bases are indicated by red dashes. E. DIC images of grhl3(-31/-31) sibling and mutant at 5 dpf. Note the defective lower law in the mutant (red arrowhead). F. Quantification of cell-debris accumulation in the chorion in grhl3 (-31/-31). (TIF) [file pgen.1009823.s009.tif]

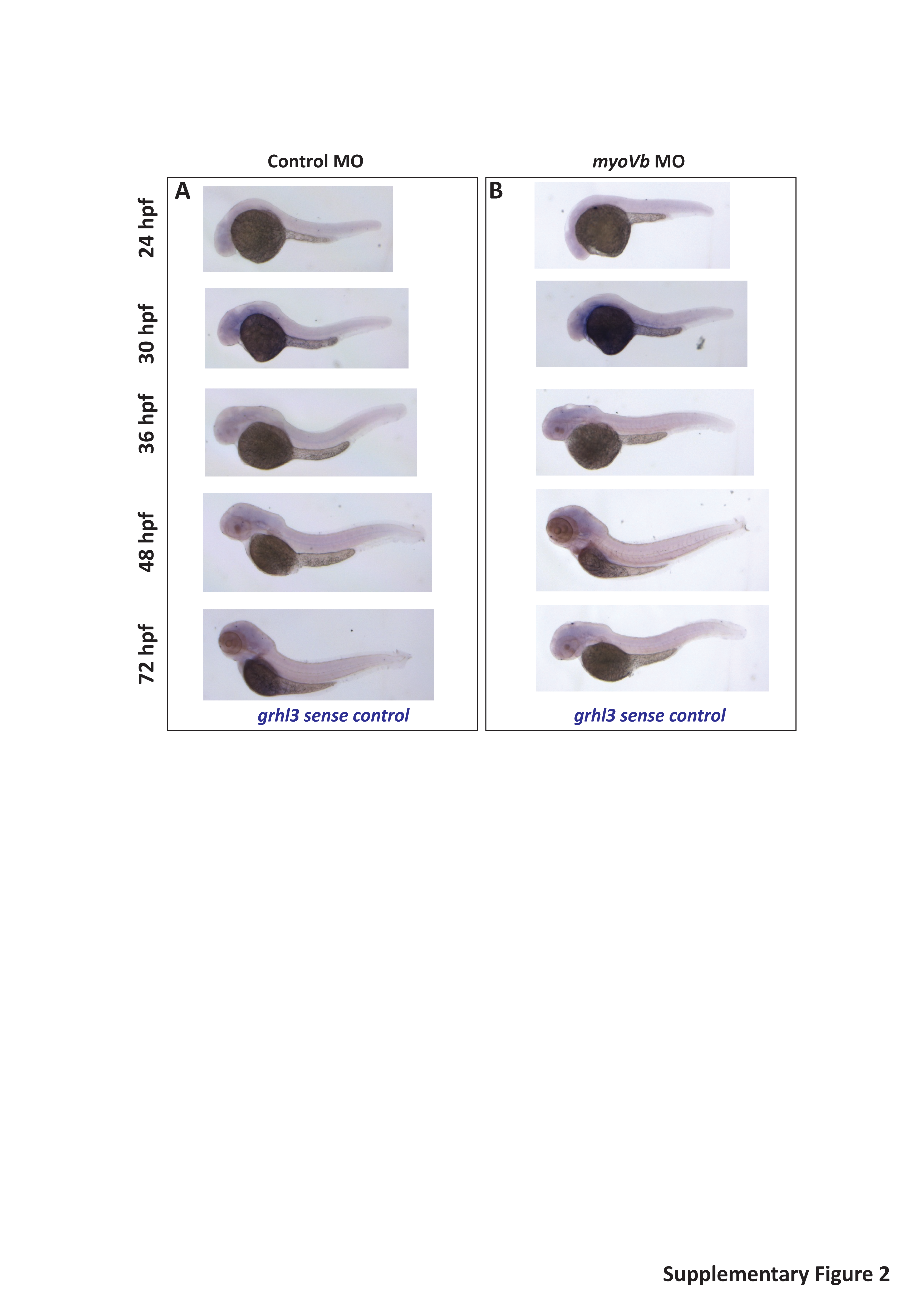

Supplement: S2 Fig — Sense controls for grhl3 in control (A) and myoVb MO embryos (B) at the given developmental time points. (TIF) [file pgen.1009823.s010.tif]

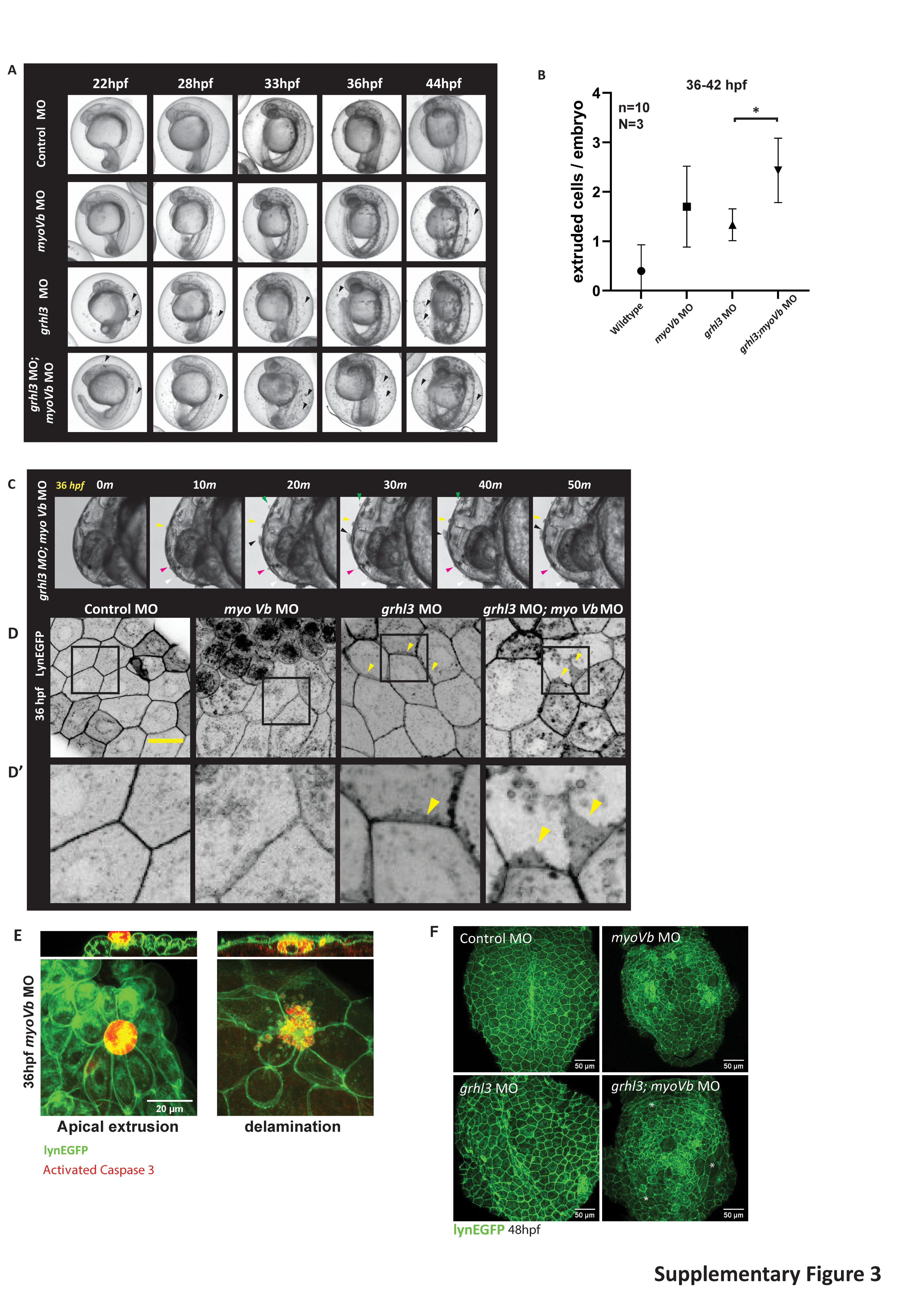

Supplement: S3 Fig — Representative stereomicroscopy images (A) of control morphants, myoVb morphants, grhl3 morphants, and myoVb;grhl3 double morphants at various time points showing progressive accumulation of cell debris (black arrowheads) in the chorion. Quantification of cell shedding using Trypan blue staining for control morphants, grhl3 morphants, myoVb morphants, and grhl3;myoVb morphants between 36 hpf and 42 hpf (B). Asterisks indicate statistically significant difference by One-way ANOVA with Tukey’s test at p<0.05. For all statistical comparisons please refer to S2 Data Representative time-lapse DIC images (C) of grhl3 MO; myoVb MO embryo mounted in E3 buffer. Colored arrowheads indicate the same extruding cells at different time-points. Representative live confocal images (D) of cldnB:lynEGFP embryos injected with control MO, grhl3 MO, myoVb MO, and grhl3 MO; myoVb MO and digitally zoomed versions (D’) of the boxed regions in D. Yellow triangles indicate profuse basolateral projections in the absence of grhl3 function, indicating weakened cell adhesion. Scale bar = 50 μm. Confocal images along with orthogonal projections of apoptotic cells, undergoing apical extrusion or delamination (E). Apoptotic cells were rarely observed. Activated caspase 3 is in Red while lynEGFP is in green. Scale bar = 20 μm. Maximum intensity projections of head periderm at 48 hpf of wild-type, grhl3 morphants, myoVb morphants, and grhl3;myoVb double morphants (F). Grhl3;myoVb morphant periderm shows a heterogeneity with patches of large and small cells. White asterisks in F indicate patches of big peridermal cells. (TIF) [file pgen.1009823.s011.tif]

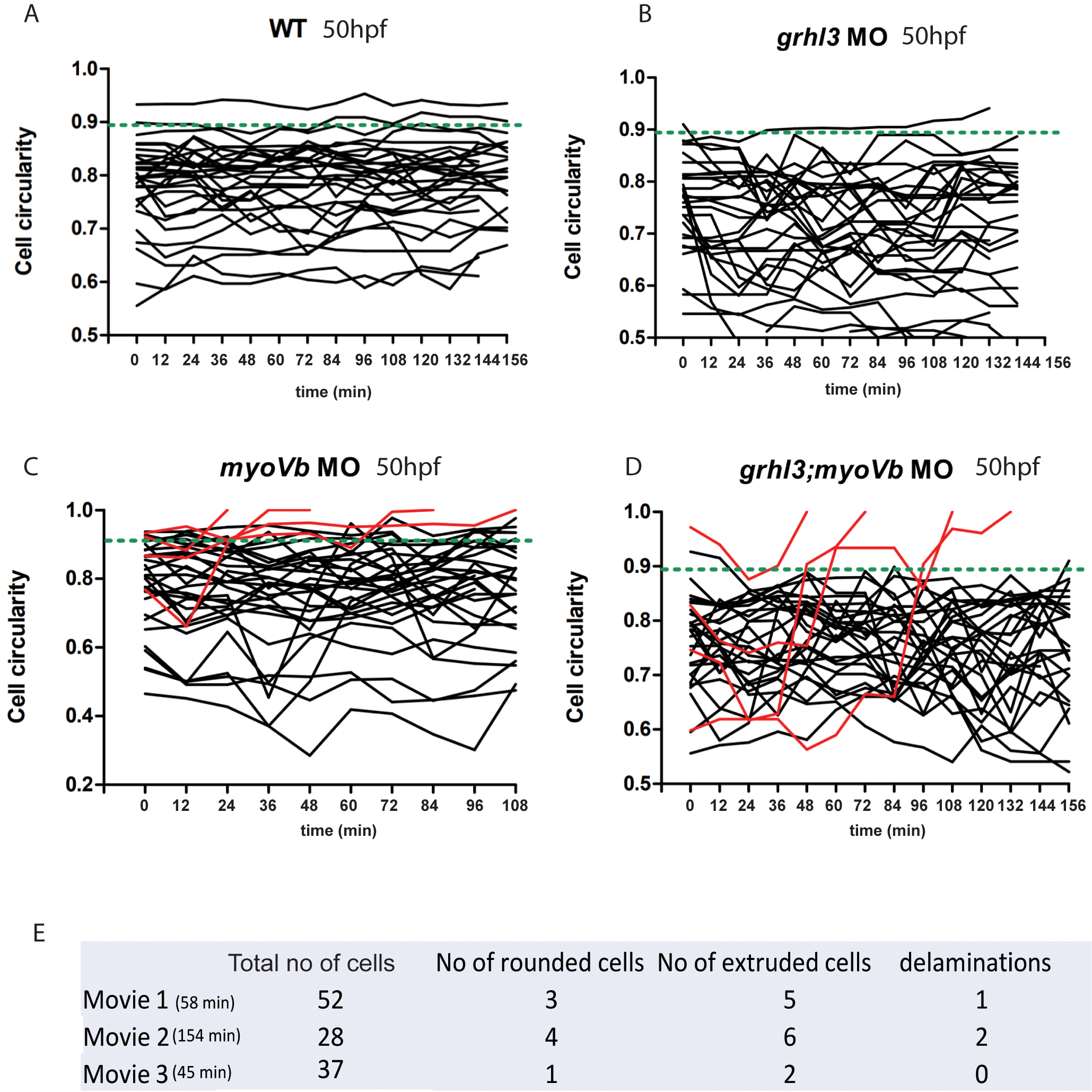

Supplement: S4 Fig — A plot of cell circularity values over time for 30 randomly selected cells (black traces) from four movies in wildtype (A), grhl3 MO (B), myoVb MO (C), and grhl3;myoVb MO (D) starting at 50hpf. Cell circularity of four extruding cells (red traces) in myoVb MO (C) and grhl3;myoVb MO (D) chosen from four movies are also shown. Green dotted line represents cell circularity threshold used for classifying rounded cells. As expected, extruding cells attain high circularity values prior to extrusion. Table E showing analysis of total number of cells and number of rounded cells, apically extruded cells and delaminated (basal cell extrusion) cells in grhl3;myoVb MO during 50 to 53 hpf. The duration of the movie is indicated in parentheses. (TIF) [file pgen.1009823.s012.tif]
